# Ketogenesis controls mitochondrial gene expression and rescues mitochondrial bioenergetics after cervical spinal cord injury in rats

**DOI:** 10.1038/s41598-021-96003-5

**Published:** 2021-08-11

**Authors:** Oscar Seira, Kathleen Kolehmainen, Jie Liu, Femke Streijger, Anne Haegert, Stéphane Lebihan, Robert Boushel, Wolfram Tetzlaff

**Affiliations:** 1 International Collaboration on Repair Discoveries (ICORD), University of British Columbia, Vancouver, BC Canada; 2 Department of Zoology, University of British Columbia, Vancouver, BC Canada; 3 Department of Biochemistry and Molecular Biology, University of British Columbia, Vancouver, BC Canada; 4 School of Kinesiology, University of British Columbia, Vancouver, BC Canada; 5 Laboratory for Advanced Genome Analysis, Vancouver Prostate Center and Department of Urologic Sciences, University of British Columbia, Vancouver, BC Canada

**Keywords:** Regeneration and repair in the nervous system, Spinal cord injury, Molecular neuroscience, Metabolism, Mitochondria

## Abstract

A better understanding of the secondary injury mechanisms that occur after traumatic spinal cord injury (SCI) is essential for the development of novel neuroprotective strategies linked to the restoration of metabolic deficits. We and others have shown that Ketogenic diet (KD), a high fat, moderate in proteins and low in carbohydrates is neuroprotective and improves behavioural outcomes in rats with acute SCI. Ketones are alternative fuels for mitochondrial ATP generation, and can modulate signaling pathways via targeting specific receptors. Here, we demonstrate that ad libitum administration of KD for 7 days after SCI rescued mitochondrial respiratory capacity, increased parameters of mitochondrial biogenesis, affected the regulation of mitochondrial-related genes, and activated the NRF2-dependent antioxidant pathway. This study demonstrates that KD improves post-SCI metabolism by rescuing mitochondrial function and supports the potential of KD for treatment of acute SCI in humans.

## Introduction

The initial tissue damage that occurs after spinal cord injury (SCI) is followed by the activation of secondary injury mechanisms, which ultimately worsen the long-term outcomes. Ischemia, excitotoxicity, inflammation and energy failure are amongst these secondary mechanisms and lead to demyelination, neuronal death, and scar formation^1–3^. Indeed, this energy failure is partially caused by impaired mitochondrial function and an increase in oxidative stress due to overproduction of reactive oxygen species (ROS)^4,5^. This increase in ROS production partly accounts for the damage caused to proteins forming the mitochondrial electron transport system (ETS), cell membranes and mitochondrial DNA^6–8^. Interestingly, an imbalance of pro- versus anti-oxidative responses to oxidative stress can reciprocally aggravate ROS production thereby increasing mitochondrial damage in a potentially vicious cycle^9^. In that regard, the acute temporal changes that affect mitochondrial bioenergetics and mitochondrial ROS production after SCI have been previously characterized^10–12^.

The ketogenic diet (KD) is a high-fat, low carbohydrate diet. The fats from the diet are converted by hepatic β-oxidation into ketone bodies which are used as alternative energy sources in the mitochondrial citric acid cycle^3,13,14^. Alternatively, the ketone body beta-hydroxybutyrate (βHB) can also act as a signaling molecule by binding to at least two cell surface receptors: HCA2 (hydroxycarboxylic acid receptor) and FFAR3 (free fatty acid receptor 3)^15–17^. While changes in signaling pathways have been identified for the niacin-mediated activation of HCA2^15,18–20^ fewer studies have looked at the effects of βHB or KD-mediated activation of HCA2^21–24^. Whether those pathways could trigger beneficial changes in the spinal cord bioenergetics after trauma is yet unknown.

Ketone bodies improve mitochondrial function in Parkinson’s, Alzheimer, amyotrophic lateral sclerosis, epilepsy and neurotrauma^17,25–32^. In fact, a growing number of publications support the idea that a shift in energy metabolism towards ketogenesis and fatty acid oxidation may play a key role in controlling mitochondrial function and mitohormesis^3^. For example, acutely after traumatic brain injury (TBI) in rats, ketones increased the expression of antioxidant enzymes preventing oxidative stress-mediated mitochondrial damage and improving mitochondrial function^33^. Furthermore, in vitro work using cortical and hippocampal neurons suggests that ketones reduce ROS production by increasing NADH oxidation^34^ through activation of mitochondrial uncoupling proteins (UCPs)^31^; and the administration of βHB rescues H_2_O_2_-mediated deficits in coupled mitochondrial respiration in rat hippocampal neurons both in vitro and in vivo^28^. In the field of SCI, we have previously shown that KD improves forelimb motor function and promotes neuroprotection in rodents after SCI^35^; and, for example, in TBI neuroprotection has been also attributed to ketones after fasting^36^. Others have shown that treatment with KD 2 weeks before cervical SCI attenuates oxidative stress by inhibition of class I HDACs^37^. Similarly, the intra-spinal treatment with D-β-hydroxybutyrate for 2 weeks reduced inflammation and O_2_ ^−^ and H_2_O_2_ formation, increased ATP formation, and improved locomotor function in mice^38^. Despite the rapidly growing research on the use of KD or ketone bodies after SCI, the mechanisms underlying its beneficial effects are not well understood.

The overall goal of the study was to elucidate the possible mechanisms by which a ketogenic state may be beneficial to counteract the mitochondrial bioenergetic dysfunction that occurs after SCI. We report that administration of KD initiated after SCI restores mitochondrial bioenergetics. Overall our data show that KD modulation of select kinases and transcription factors involved in the control of the cellular and mitochondrial transcriptional and translational machineries together with the activation of anti-oxidant defence mechanisms globally contribute to the rescue of mitochondrial bioenergetics after cervical spinal cord injury.

## Results

### Ketogenic diet induces ketosis in rats 7 days after SCI

**Figure 1:**
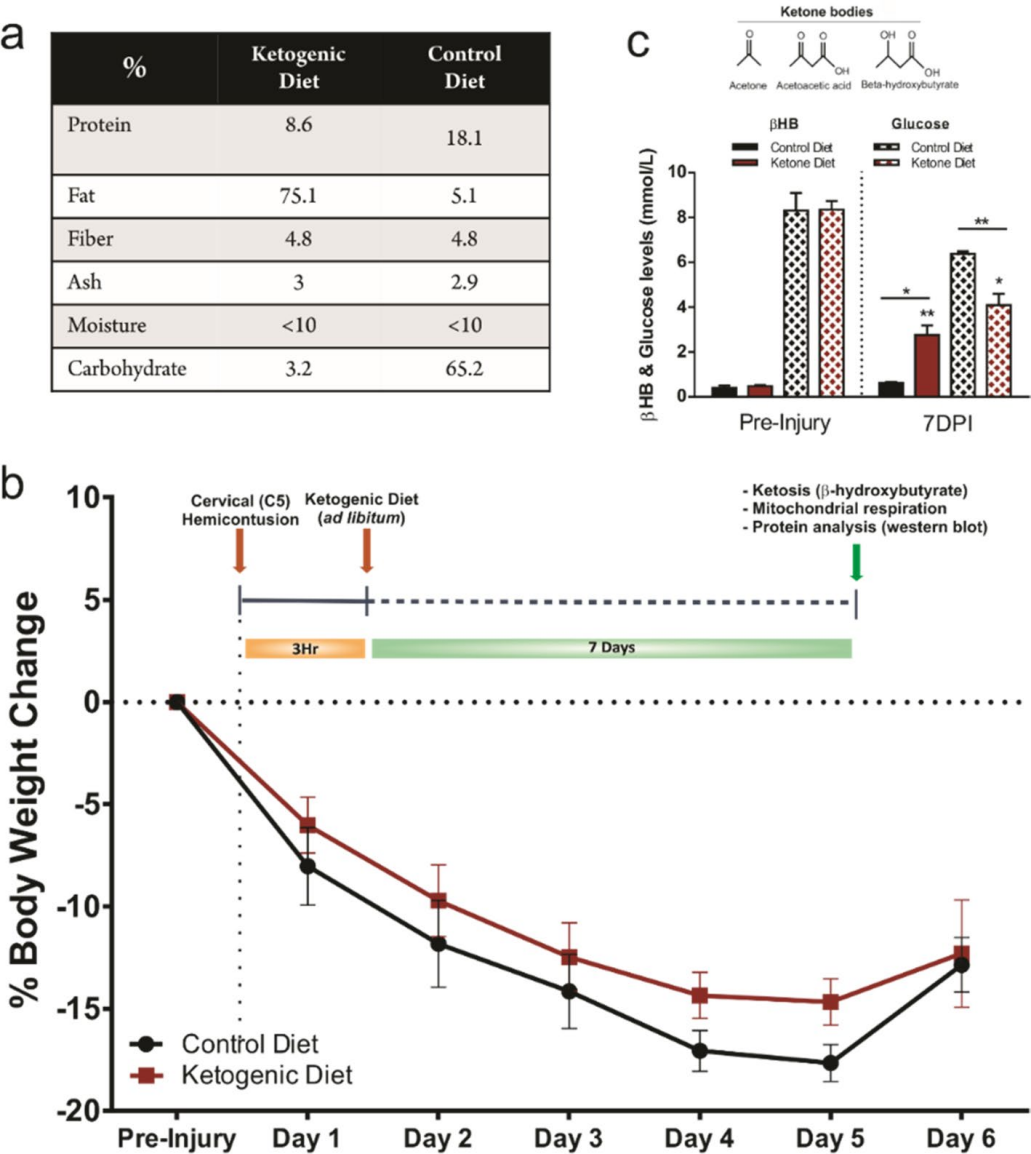
(**a**) Table showing the control and ketogenic diets breakdown (%) (CD: F5960, Bioserv; KD: F5848, Bioserv). (**b**) Experimental timeline and percentage weight change along the experiment. Data are the means ± SEM. **c)** Blood ketone (β-hydroxybutyrate) levels increased and glucose levels decreased when consuming KD ad libitum. *Data are the means* ± *SEM. Student’s t-Student test.*

We first examined the effectiveness of the Bioserv F5848 ketogenic diet (KD) in inducing ketosis in rats. This KD formulation (Fig. 1a) led to a significant increase in blood β-hydroxybutyrate levels one week post SCI while at the same time glucose levels were significantly reduced (Fig. 1c). As expected, a significant weight loss occurred during the first days post-injury (DPI). However, the animals’ weights stabilized and recovered starting at 5 and 6 DPI. Interestingly, the animals that were fed with KD showed a trend towards a better weight recovery 4 and 5 DPI when compared to the animals fed with the control diet (CD). Weights from both groups were equal by 6 DPI (Fig. 1b).

### Ketogenic diet rescues complex I and II-driven respiration, improves complex IV activity and increases complex I, II and III protein expression following SCI

**Figure 2:**
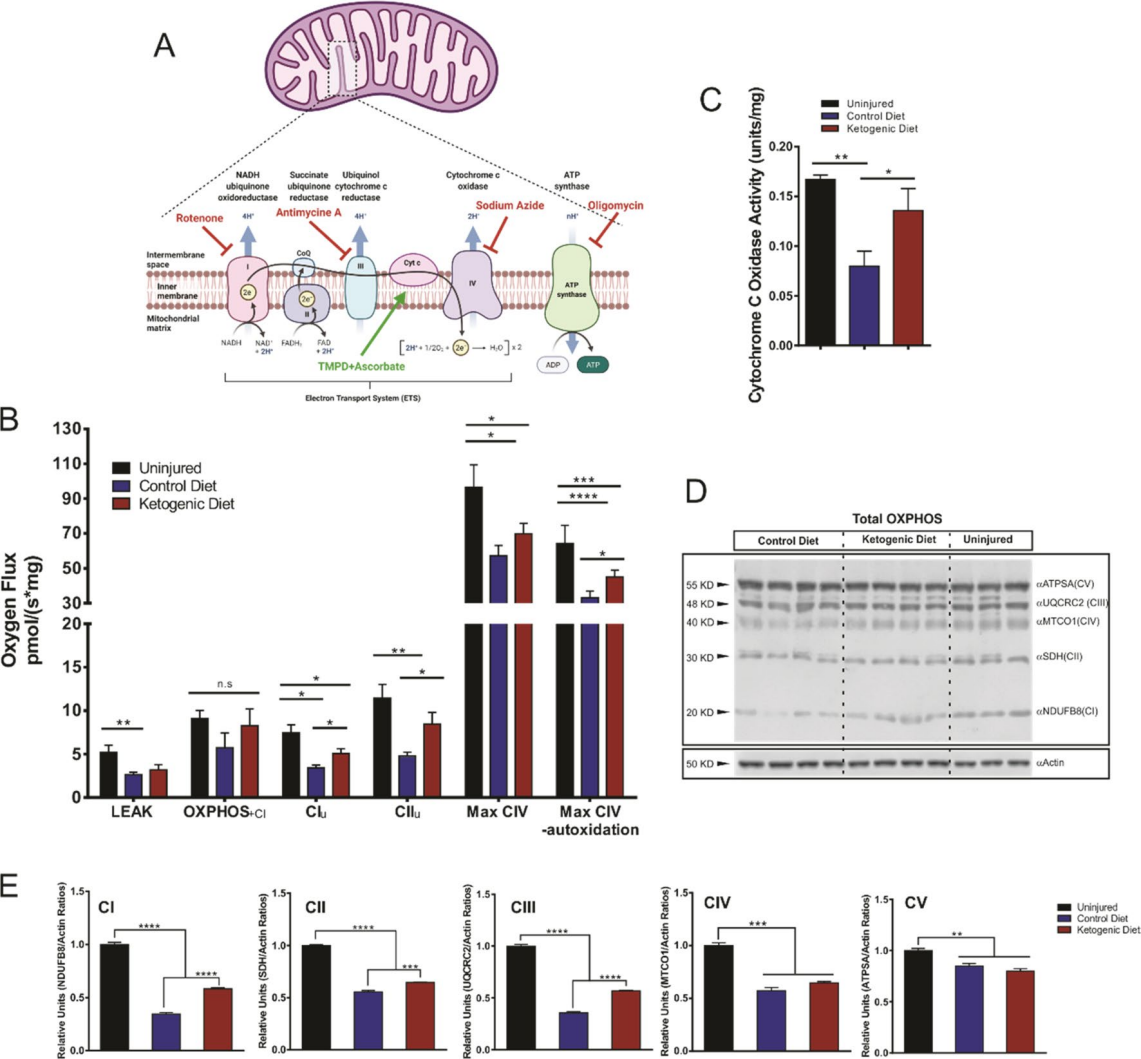
Mitochondrial respiration and western blotting analysis of the relative levels of the 5 OXPHOS complexes. (**A**) Schematic representation of the mitochondrial Electron Transport System (ETS). *Adapted from “Electron Transport Chain”, by BioRender.com (2020). Retrieved from *https://app.biorender.com/biorender-templates*.* (**B**) Quantification of mitochondrial respiration by high-resolution respirometry showing Oxygen flux values of the respiratory states. *OXPHOS*: Representing the maximum oxidative phosphorylation in the presence of metabolic substrates after the addition of ADP; *LEAK respiration (proton leak)*: Mitochondrial respiration rate in the presence of an ATP synthase inhibitor (Oligomycin); *Complex I (CI) Maximum respiration*: Maximum respiration measured after addition of the uncoupler carbonyl cyanide m-chlorophenyl hydrazone (CCCP) causing a depolarization of the cell membrane and leading to an increase in respiration; *Complex II (CII) Maximum respiration*: Maximum respiration measured after inhibition of CI with Rotenone and the subsequent addition of its substrate, Succinate; *Complex IV (CIV) Maximum respiration*: Maximum respiration estimated after inhibition of Complex III with Antimycin A, and the subsequent addition of ascorbate and N,N,N′,N′-tetramethyl-p-phenylenediamine (TMPD) as proton donor substrates. Finally, NaN3 (Sodium Azide) was added to block the respiratory chain by blocking ATPase and leaving the fraction of total respiration that is non-mitochondrial, this value is subtracted from the Complex CV Maximum respiration in order to estimate the real mitochondrial CIV respiration*. Student’s t-Student tests*. (**C**) Cytochrome C Oxidase (or Complex IV) activity assay shows an increase in activity in the KD treated group. (**D**,**E**) Western blot of total OXPHOS proteins and subsequent quantifications showing an increase in the protein levels in CI, CII and CII after KD treatment versus the CD. *One-way ANOVA, Tukey’s Post-hoc test. All data are mean* ± *SEM.*

To evaluate the possible benefits of KD on energy metabolism following SCI we studied mitochondrial respiratory function and oxidative phosphorylation (OXPHOS) in the injured cervical spinal cord segments (Fig. 2a). For this, we used a substrate-uncoupler-inhibitor-titration protocol (see “Methods”) based on a previous report by Sullivan et al.^10^. As expected, our respirometry results showed an overall decline in mitochondrial respiration (oxygen consumption) at 7 DPI. Following SCI, mitochondrial respiration measured in the presence of malate, pyruvate, glutamate, succinate and the uncoupler CCCP showed a significant decrease in Complex I (CI_u_) and Complex II (CII_u_) respiration (Fig. 2b). LEAK respiration measured after oligomycin addition to block ATP-synthase activity was significantly decreased after injury in both treated groups, CD and KD. The decrease in LEAK after injury was not prevented with the KD treatment (Fig. 2b). The ADP-stimulated OXPHOS for complex I showed greater variability and the apparent differences observed between the uninjured group and the injured groups (CD nor KD) did not reach significance. However, in the KD group, mitochondrial respiration with convergent electron supply through Complex I and Complex II were significantly higher compared to the CD group (48.12% increase, p = 0.028 for CI; 76.07% increase, p = 0.025 for CII). Mitochondrial respiration via Complex IV (CIV) also showed a significant increase in the KD treated group after the complex was fully inhibited with sodium azide in order to control for oxygen consumption through autoxidation (or spontaneous oxidation) of tetramethylphenylenediamine (TMPD) (35.82% increase, p = 0.0469) (Fig. 2b). Although respiratory rates and control ratios were somewhat low in the permeabilized spinal cord tissue compared to other tissues, previous literature reveals variability in the rates between different models, tissues and cell types. The finding that respiration with addition of the uncoupler CCCP showed only modest increases above coupled respiration indicates that tissues were sufficiently permeabilized.

To confirm the increase in cytochrome c oxidase (Complex IV) respiration, we decided to perform a colorimetric enzymatic assay in tissue homogenates from a second cohort of animals with the exact same injury and treatment conditions. Using this enzymatic approach we also saw significant changes in cytochrome c oxidase activity between treatment groups (Fig. 2c). The decrease in cytochrome c oxidase activity after injury (52.17% decrease, p = 0.087) was reversed to almost uninjured activity levels in the animals treated with KD (Fig. 2c). Similar results were observed when cytochrome c oxidase activity was measured in isolated mitochondria (See “Methods” section) from the epicenter of the injury (Supplementary Fig. S4).

Additionally, in order to identify changes in protein expression levels of the ETS complexes after injury and dietary treatment, a western blot analysis using a “total OXPHOS” antibody cocktail was performed in tissue homogenates obtained from the epicenter of the injury (Fig. 2d,e). These blots revealed an overall significant decrease in protein content at 7 DPI. Interestingly, the cords from the KD treated group showed a significant rescue in expression of complexes I, II and III compared to CD (68.8% increase, p < 0.001 for CI; 16.36% increase, p = 0.003 for CII; and 60.12% increase, p < 0.001) (Fig. 2d,e). No significant differences were observed in the protein content of complexes IV and V between the CD or KD groups, although there was a trend towards increased complex IV expression with KD (p = 0.0748) (Fig. 2d,e).

### Changes in mitochondrial markers after ketogenic diet treatment

**Figure 3:**
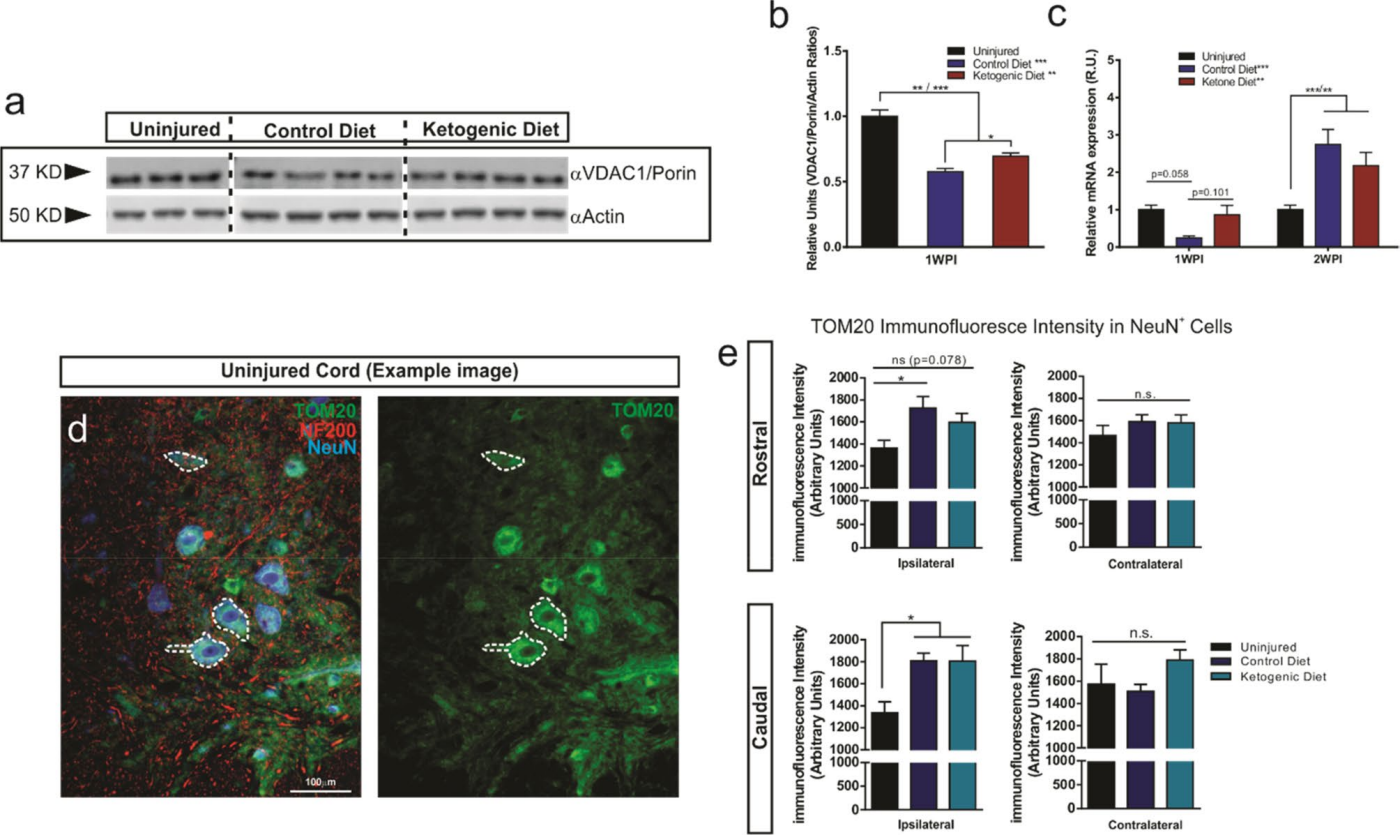
Expression of mitochondrial markers after SCI. (**a**,**b)** Western blot and quantification using an antibody against VDAC1/Porin, an outer mitochondrial ion dependent channel. *One-way ANOVA, Fisher’s LSD Post-hoc test. All data are mean* ± *SEM*. (**c**) Relative gene expression of Peroxisome proliferator-activated receptor gamma coactivator 1-alpha (PGC-1α). The expression levels of mRNAs were measured by real-time PCR. Values represent relative expression levels of PGC1-α in uninjured rats (n = 5) compared to the injured spinal cord of KD rats (n = 6) compared with CD rats (n = 6). *Two-way ANOVA, Tukey’s Post-hoc test. All data are mean* ± *SEM.* (**d**,**e**) Example high magnification micrograph of the ventral horn of an uninjured spinal cord stained with TOM20 (mitochondria), NF200 (Neurofilament) and NeuN (D). Intensity quantifications of the mitochondrial import receptor subunit (TOM20) from randomly outlined NeuN^+^ cells (n = 9–12) (**e**). *One-way ANOVA, Fisher’s LSD Post-hoc test. All data are mean* ± *SEM.* Two different mitochondrial markers both targeting outer mitochondrial membrane proteins were used due to its differential effectiveness in detecting the target in western blots or in immunofluorescence. *The blots from the same gels were cropped and rearranged in the following order: Uninjured, Control Diet, Ketogenic Diet; to make them more clear and enhance reader’ understanding of the figure and the subsequent quantifications.* Membranes were cut previous incubation with primary antibodies in order to optimize limited sample use. *Full-length blots/gels are presented in Supplementary Fig. 5.*

We next asked whether KD rescues mitochondrial function via activation of the mitochondrial biogenesis PGC1α-SIRT3-UCP2 axis. An increase in this axis by KD was recently shown in hippocampal neurons *in vitro*^28^. We analyzed the expression of voltage-dependent anion selective channel protein 1 (VDAC1/Porin) and peroxisome proliferator-activated receptor γ-coactivator-1α (PGC-1α) by western blot and RT-qPCR respectively (Fig. 3). Our western blot analysis showed a significant decrease in VDAC1 content in the injured spinal cords in both CD and KD groups one week post-injury (Fig. 3a,b). Interestingly, treatment with KD significantly increased VDAC1 expression when compared to the CD treated group (20.71% increase, p = 0.0217). Furthermore PGC-1α mRNA levels showed a trend towards an increase in the KD animals compared to the CD treated animals at 7DPI (p = 0.101). This trend that was no longer seen at 2-weeks post-injury, suggesting that the possible effect of KD may be transient or time-dependent (Fig. 3c). Interestingly, our 2 weeks post-injury data also show a significant upregulation in both injured groups (Fig. 3c). In addition, using a histological approach, we wanted to evaluate if changes in expression of mitochondrial markers were taking place in neurons located in the vicinities of the injury site rostral and caudal to the injury (Fig. 3d,e). Interestingly, we observed a significant increase in the mitochondrial import receptor subunit TOM20 (TOM20) immunofluorescence mean intensity in NeuN^+^ cells both rostral and caudal to the injury site (ipsilateral) when compared to the uninjured spinal cords (25% increase rostral, p = 0.0185 (CD); ~ 32% increase caudal, p = 0.048 (CD) p = 0.032 (KD)) . No difference between CD and KD treated groups was found. The contralateral side (non-injured) did not show differences between any of the three groups (Fig. 3d,e).

### Transcriptional and translational regulation by ketogenic diet after SCI

**Figure 4:**
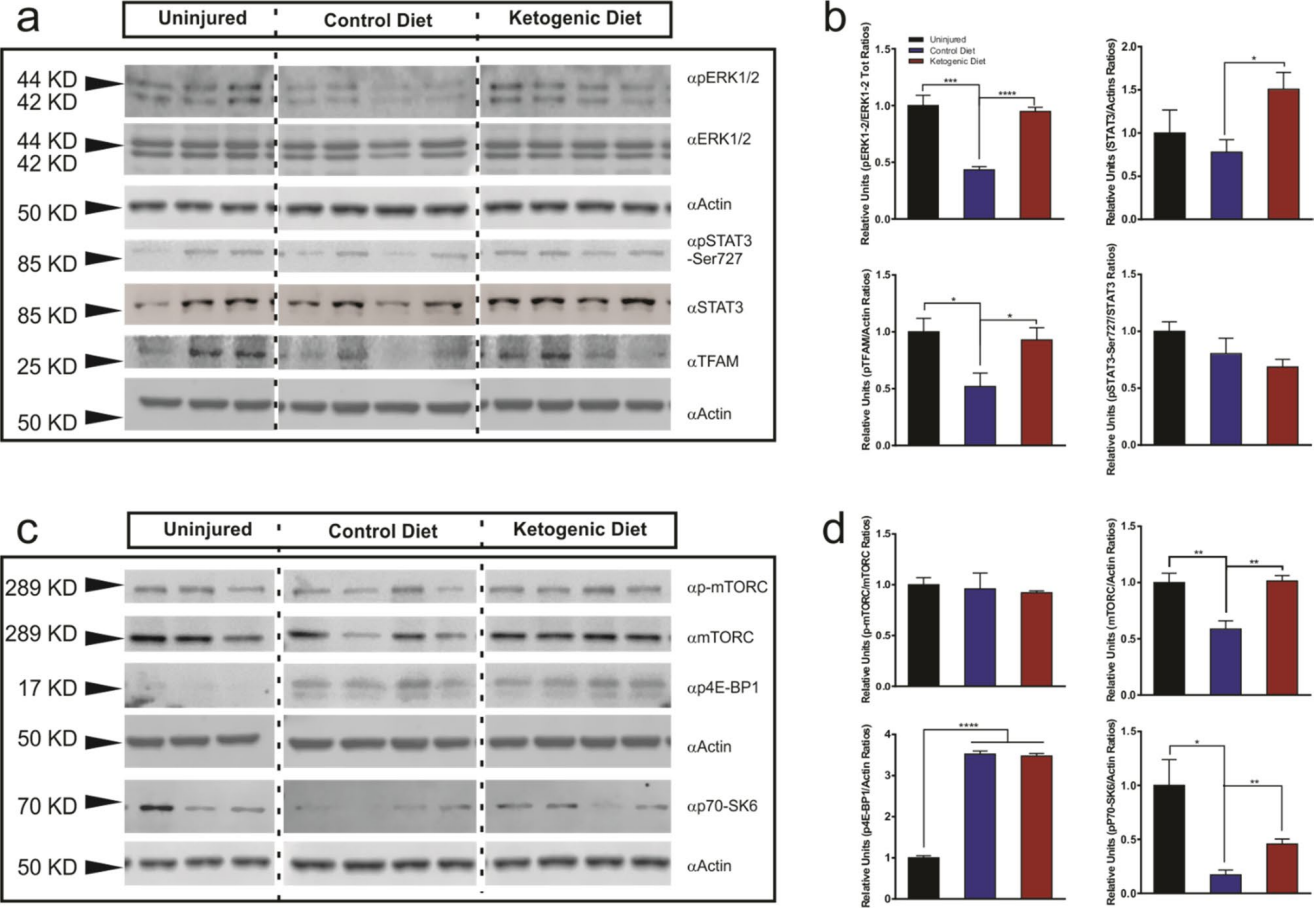
Western blot analysis of mTFAM and members of the MAPK, mTOR and STAT signaling pathways after KD treatment in spinal cord tissue homogenates. (**a**) Immunoblots showing an increase in ERK1/2 phosphorylation at the Thr42/44 epitope, increased mTFAM and total Stat3 expression in injured spinal cords after KD treatment. (**b**) Quantifications of blots in (**a**). (**c**) Immunoblots showing an increase in p70-S6K phosphorylation at the Thr202/Tyr204 epitope and an increase in total mTOR expression in injured spinal cords after KD treatment. (**d**) Quantifications of blots in (**c**). *One-way ANOVA, Fisher’s LSD Post-hoc test. All data are mean* ± *SEM. The blots from the same gels were cropped and rearranged in the following order: Uninjured, Control Diet, Ketogenic Diet; to make them more clear and enhance reader’ understanding of the figure and the subsequent quantifications.* Membranes were cut previous incubation with primary antibodies in order to optimize limited sample use. *Full-length blots/gels are presented in Supplementary Fig. 5.*

We further analyzed whether the treatment with KD could regulate transcription factors and proteins associated with the general control of the transcriptional and translational machineries in the injured spinal cord (Fig. 4). Using spinal cord homogenates, we first examined p44/42 MAPK (ERK1/2) and STAT3. Both kinases appear to play central roles in mitochondrial regulation and it has been suggested that they are translocated to the mitochondria and regulate the transcription of mtDNA^39,40^. Western blot analysis showed a significant decrease in phospho-p44/42 MAPK (ERK1/2) (Thr202/Tyr204) after SCI (56.6% decrease, p = 0.001) (Fig. 4a,b). The phosphorylation analysis of ERK1/2 revealed a significant increase in phosho-ERK1/2 in the KD group compared to the CD group (118.52% increase, p < 0.0001) (Fig. 4a,b). A significant increase in total STAT3 expression was observed in the KD group compared to the CD group (93.22% increase, p = 0.026). However, no significant changes in STAT3 phosphorylation levels after SCI nor between treatment groups was detected (Fig. 4a,b).

Next, we examined the phosphorylation levels of mTORC and two of its well-known downstream effectors: 4E-BP11 and p70-S6K. Amongst many other roles, these kinases also regulate the nutrient and growth factor protein biosynthesis in response to nutrient stimuli^41^. Furthermore, ketone bodies have been shown to induce mTORC inhibition in the hippocampus of rats fed with KD^42^. While a significant decrease in total mTORC content was observed after injury and this decrease was rescued by KD, we saw no differences in mTORC phosphorylation levels between KD and CD-treated groups (Fig. 4c,d). Despite no changes in mTORC activation after injury, we did see changes in its downstream effectors. After SCI, the phosphorylation of the translation initiation factor 4E-BP1 was remarkably increased in both CD and KD groups (253% increase, p < 0.0001). However, no differences were observed between the treatments (Fig. 4c,d). In contrast, the serine/threonine kinase p70-S6K showed a considerable decrease in phosphorylation on Thr389 epitope after injury (82.91% decrease, p = 0.0102) (Fig. 4c,d), and its phosphorylation was significantly increased after KD treatment when compared to the injured group treated with the CD (167.38% increase, p < 0.005) (Fig. 4c,d).

We further studied the expression of the mitochondrial transcription factor A (TFAM). TFAM is a nuclear DNA-encoded protein that binds to the mtDNA and promotes transcription^43^. Our data show that in tissue homogenates TFAM was significantly downregulated after injury (48.14% decrease, p = 0.037); the treatment with KD upregulated its expression to nearly uninjured levels (78.92% increase, p = 0.04) (Fig. 4a, b).

### Modulation of intrinsic mitochondrial components by ketogenic diet after SCI

In order to study changes in the mitochondrial intrinsic signaling pathways after KD treatment, mitochondrial enriched fractions from spinal cords were isolated by gradient centrifugation. Samples were analyzed by western blot and normalized to total VDAC1 content (Supplementary Fig. S2a,b).

Phosphorylation of mitochondrial ERK1/2 was significantly increased after SCI (86% increase, p < 0.01) (Supplementary Fig. S2c,d). Although we did not see significant differences between treatment groups, the KD treated group showed a slight trend towards a decrease in phosphorylation compared to the CD group (Supplementary Fig. S2c,d). STAT3 showed an overall increase in Ser-727 phosphorylation after injury. A significant increase in STAT3 activity was seen after KD treatment when compared to the injured group and the CD treated group (205.1% increase, p < 0.001 and 57.18% increase, p = 0.0294 respectively) (Supplementary Fig. S2c,d). In isolated mitochondria, mTORC phosphorylation was not different between groups, consistent with the data obtained from the tissue homogenate samples (Supplementary Fig. S2c,d). In contrast, when we examined total protein content, total mTORC protein was remarkably higher after injury in the CD group compared to the uninjured group (162.2% increase, p < 0.01), while its expression levels after KD treatment were reduced to levels that were comparable to the ones found in the uninjured group (Supplementary Fig. S2c,d). The phosphorylation levels of p70-S6K followed a similar pattern of phosphorylation to that seen with ERK1/2: a significant increase in phospho-p70-S6K was observed after injury (88.5% increase, p = 0.01) and a trend towards a reduction in phosphorylation was seen in the KD treated group (p = 0.07) (Supplementary Fig. S2c,d). Similar to the mTORC data from isolated mitochondria, these results differ from those observed in the tissue homogenate samples. Furthermore, our western blot analysis showed a significant increase in TFAM expression in the mitochondria that were isolated from the cords treated with KD.

A mitochondrial population of NRF2 has been described that forms a complex containing a KEAP1 dimer and the mitochondrial histidine phosphatase PGAM5^44,45^. Recent data also suggest that NRF2 might protect mitochondria from oxidative damage through direct interaction with the mitochondrial outer membrane^46^. Interestingly, our results showed a significant reduction in the levels of phosphorylated NRF2 in isolated mitochondria after injury (69.7%, p = 0.0131); after KD treatment we observed a trend towards a partial rescue (p = 0.13) (Supplementary Fig. S2c,d). These results point out the importance of studying the different protein pools and populations from different organelles and compartments separately.

We further analyzed the effect of KD in modulating the expression of the ETS complexes in isolated mitochondria. Similar to the tissue homogenates, we saw an overall decrease in expression of Complexes I to IV after SCI (Supplementary Fig. S2e,f). However, contrary to the tissue homogenate results, no statistical differences were found in the expression levels between CD and KD treated samples. Even so, trends towards an increase in expression in the KD group can be seen for CI and CII (Supplementary Fig. S2e,f).

### Ketogenic diet reduces oxidative damage and induces activation of the NRF2-dependent antioxidant pathway after SCI

**Figure 5:**
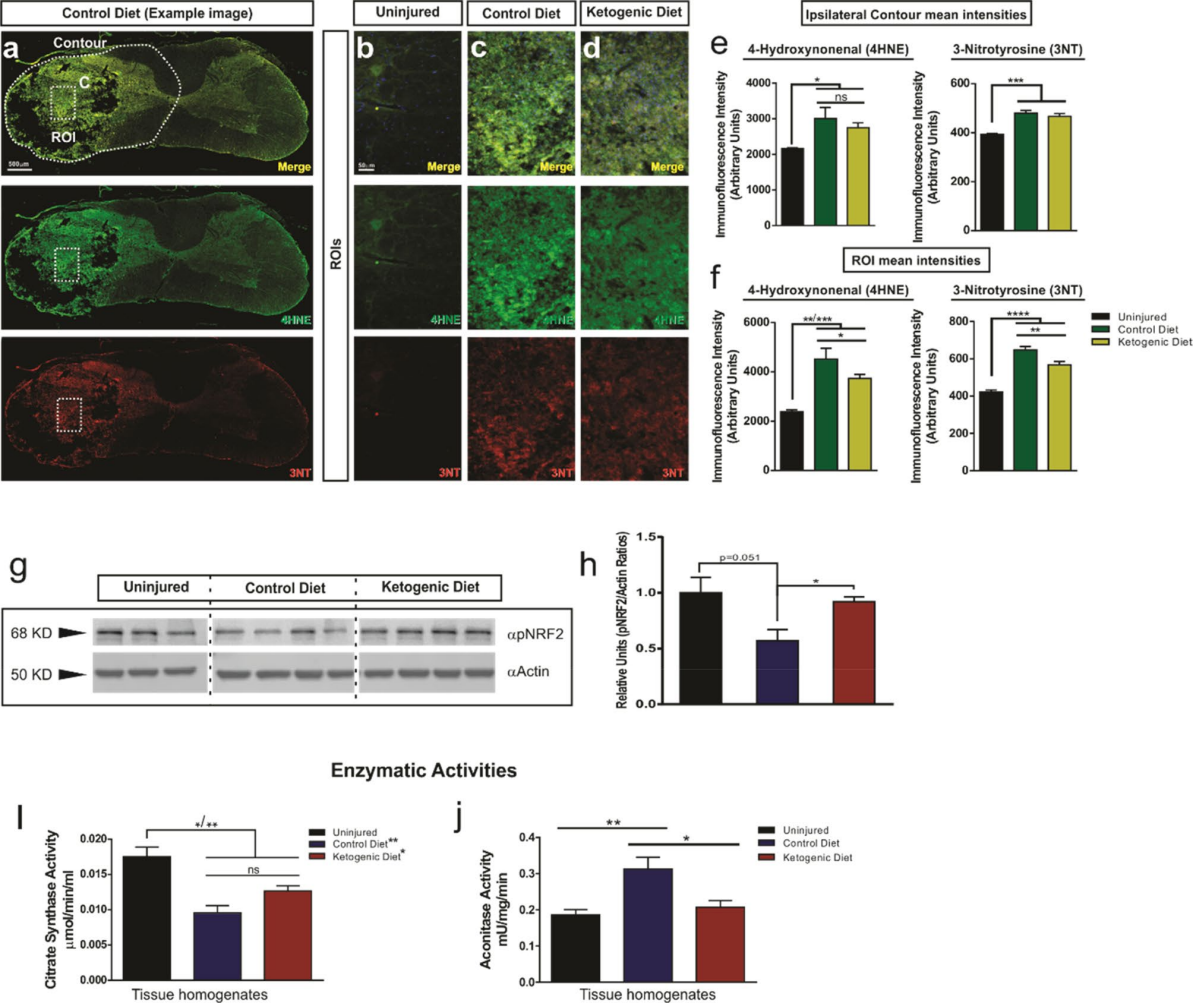
Effect of KD and βHB treatments on oxidative stress in vivo. (**a–f**) Double immunostainings of the spinal cord with the lipid peroxidation marker 4HNE and the protein nitration marker 3NT. Low–power example image of a cross section of an injured cord treated with CD (**a**). Dashed white outline (contour) and square (ROI) showing an example of quantified areas in (**e**–**f**). (**b–d**) High magnification example images of the ROIs selected form the epicenter of the injury from uninjured, and injured cords after CD or KD treatment. Intensity quantifications of the ipsilateral contour and ROI of the epicenter of the injury are shown in (**e**,**f**). Uninjured (n = 4), control diet, (n = 4) and ketogenic diet (n = 6); *One-way ANOVA, Fisher’s LSD Post-hoc test. Values are shown as mean* ± *SEM*. (**g**,**h**) Spinal cord homogenates were subjected to SDS-PAGE followed by western blot analysis using phospho-NRF2 antibody. Quantification is shown in (**h**). (**i,j**) Citrate synthase (CS) and Aconitase (AC) activities were measured in spinal cord tissue homogenates from uninjured (n = 3), control diet, (n = 4) and ketogenic diet (n = 4) treated animals. The epicenter of the injury was used for the analysis in the animals with SCI. *The blots from the same gels were cropped and rearranged in the following order: Uninjured, Control Diet, Ketogenic Diet; to make them more clear and enhance reader’ understanding of the figure and the subsequent quantifications.* Membranes were cut previous incubation with primary antibodies in order to optimize limited sample use*. Full-length blots/gels are presented in Supplementary Fig. 5. One-way ANOVA, Fisher’s LSD Post-hoc test. Values are shown as mean* ± *SEM.*

ROS formation after trauma contributes to the pathogenesis associated with SCI by affecting the mitochondrial respiratory system itself, and by inducing lipid peroxidation and protein nitration. Lipid peroxidation and protein nitration has long been known as a main contributor to secondary damage after SCI and it has been shown to increase after injury^47–49^. Our histological data showed an increase in lipid peroxidation and protein nitration using 4HNE and 3NT markers respectively at 7 days post injury (Fig. 5a–f). Interestingly, when we compared the immunofluorescence mean intensity between treatment groups, we observed a significant reduction of both markers in the KD group compared to the CD group in the center of lesion (ROI) (17.2%, p = 0.046 (4HNE); 12.3%, p = 0.0047 (3NT)) area with most of the infiltrating immune cells (Fig. 5a–d,f). However, this reduction was no longer significant when the intensity of the entire contour of the ipsilateral side of the cord was measured, which included a large area with low signal (Fig. 5a,e).

ROS can also trigger activation of specific intracellular antioxidant pathways as a protective response to injury^50^. In order to assess the activation of intracellular antioxidant mechanisms after KD treatment, we used Western blots to quantify levels of phosphorylated (active) NRF2, an upstream transcription factor that regulates the expression of several antioxidant genes (Fig. 5g,h) in tissue homogenates. Our results showed that while the level of active NRF2 decreased after SCI (p = 0.051), those levels were fully rescued by KD (Fig. 5g,h).

### β-hydroxybutyrate (βHB) increases NRF2 activation after H_2_O_2_-induced injury and downregulates p21 in non-H_2_O_2_-induced injury conditions in cortical neurons

β-hydroxybutyrate (BHB) is the most abundant ketone body found in the blood as a result of exogenous KD ingestion^51^. We used in vitro cultures of primary cortical neurons to evaluate whether β-hydroxybutyrate (BHB) alone would be sufficient in promoting an increase in NRF2 activity in response to cellular ROS production. Hence, H_2_O_2_ (10 μM) was added to the culture media to induce oxidative stress and initiate an antioxidative response. The cultures were incubated with BHB (4 mM) alone or in combination with H_2_O_2_ for 24 h (Supplementary Fig. S3a). Our immunofluorescence intensity analysis showed that addition of H_2_O_2_ increases NRF2 activity—as shown by an increase in Serine 40 (S40) phosphorylation (162%, p < 0.0001) (Supplementary Fig. S3b,d,g). Interestingly, when compared to untreated cultures exposed to H_2_O_2_ alone, the H_2_O_2_ exposed cultures treated with BHB showed an even higher increase in S40 phosphorylation (216.9%, p < 0.0001) (Supplementary Fig. S3b,e,g). The treatment with BHB alone had no effect on pNRF2 levels (Supplementary Fig. S3c,g). Moreover, to further assess the effect of KD on regulation of oxidative stress, we evaluated p21 intensity levels. p21 is a cyclin dependent kinase inhibitor (CDK) that regulates multiple cellular processes such gene transcription, cell differentiation and apoptosis. p21 has been also associated with a new protective role against oxidative stress in cells^52,53^. Our results showed that p21 expression in cortical neurons was significantly downregulated after BHB treatment (25%, p < 0.001) (Supplementary Fig. S3c,f). Cortical cultures treated with H_2_O_2_ showed a dramatic decrease in p21 immunofluorescent intensity (83%, p < 0.0001); this decrease was not prevented with addition of BHB (4 mM) (Supplementary Fig. S3d–f).

### Ketogenic diet alters Citrate synthase activity and decreases Aconitase activity after SCI

The activities of the mitochondrial enzymes aconitase (AC) and citrate synthase (CS) were evaluated after SCI as previous work linked AC and CS activity to the regulation of the cellular redox state in response to ROS production^54–57^. Furthermore, it has already been established that ROS production increases after SCI^10,33^. Therefore, we analyzed the enzymatic activities of CS and AC in spinal cord homogenates after injury and treatment with KD (Fig. 5i,j). Our activity assay showed a significant decrease in CS activity after injury in the animals that were fed CD (45.37%, p < 0.01). There was a trend (p = 0.11) towards an increase in CS activity with KD treatment. (Fig. 5i). Using a different cohort of animals, we measured CS activity of isolated mitochondria from the injury site. Contrary to what we observed in tissue homogenates, CS activity in isolated mitochondria showed a significant decrease in activity in the KD treated group, but no differences were found between the CD group and the uninjured nor the KD group (Supplementary Fig. S4). After SCI, AC activity increased significantly in the CD fed group (68.18%, p < 0.01) (Fig. 5j) and KD treatment reduced AC to nearly uninjured levels (Fig. 5j).

### Effects of ketogenic diet on expression levels of mRNAs encoding proteins involved in mitochondrial metabolic and intracellular signaling pathways

RNA from the injured spinal cords was analyzed from rats fed either a KD or a CD for a week. The microarray analysis indicated significant changes (p < 0.05) in gene expression for a total of 1018 genes (530 increased; 488 decreased). The data for all genes detected as certain signals were deposited in the NCBI Gene Expression Omnibus (GEO, http://www.ncbi.nlm.nih.gov/geo/) under accession number GSE159555. Amongst those genes, in Table 1 we show a subset of significantly increased or decreased genes that includes genes associated with mitochondria, transcription and translation, metabolic pathways and oxidative stress responses. We found increased levels of mitochondrial-specific mRNAs that either encode for different subunits of complexes of the ETS (*Ndufa12, Nda6, Atp5I*), or that encode for proteins associated with the assembly or synthesis of its components (*Coq3* (p = 0.055) and *Cox11*). Moreover, the microarray showed increased and decreased expression of mitochondrial ribosomal proteins such *Mrps14* and *Mrpl22* (up) *and Ptcd3* (down); increase levels of *Pdk4* that encodes for the pyruvate kinase responsible for inhibiting pyruvate dehydrogenase, regulation of glucose-fatty acid homeostasis, and an increase in the RNA encoding the outer mitochondrial membrane anti-apoptotic factor *Bcl2*.

**Table 1:** List of genes showing significant fold change in microarray.

Gene	Gene name	Fold change (Absolute) KD vs CD	P-value
**Upregulated**			
Mitocondrial associated genes			
*Coq3*	Hexaprenyldihydroxybenzoate methyltransferase (Synthesis of Coenzyme Q)	1.8260276	p = 0.055436
*Nd6*	NADH-ubiquinone oxidoreductase chain 6 (Complex I)	1.2989544	0.028589
*Pdk4*	Pyruvate dehydrogenase kinase, isozyme 4	1.2670953	0.028164
*Cox11*	COX11 cytochrome c oxidase assembly homolog (yeast) (Assembly of Complex IV)	1.243264	0.03916
*Bcl2*	B-cell CLL/lymphoma 2	1.2251029	0.009542
*Ndufa12*	NADH dehydrogenase (ubiquinone) 1 alpha subcomplex, 12 (Complex I)	1.2011336	0.014646
*Tomm40b*	Translocase of outer mitochondrial membrane 40 homolog B (yeast)	1.1915064	0.020061
*Mrps14*	Mitochondrial ribosomal protein S14	1.147669	0.015547
*Gcsh*	Glycine cleavage system protein H (aminomethyl carrier)	1.116142	0.029307
*Mrpl22*	Mitochondrial ribosomal protein L22	1.1113199	0.018437
*Ssbp1*	Single-stranded DNA binding protein 1	1.1083369	0.035728
*Atp5l*	ATP synthase, H + transporting, mitochondrial F0 complex, subunit G (Complex V)	1.1060036	0.019063
Transcription, translation and metabolism associated genes			
*Mttp*	Microsomal triglyceride transfer protein	1.7323127	0.015456
*Fads6*	Fatty acid desaturase domain family, member 6	1.5089704	0.0251
*Pank1*	Pantothenate kinase 1	1.2655075	0.044121
*Nfyb*	Nuclear transcription factor-Y beta	1.2637761	0.016081
*Polr2f*	Polymerase (RNA) II (DNA directed) polypeptide F	1.2126642	0.038375
*Apoo*	Apolipoprotein O	1.2104626	0.015709
*Tceal1*	Transcription elongation factor A (SII)-like 1	1.163035	0.013797
*Cyp2d4*	Cytochrome P450, family 2, subfamily d, polypeptide 4	1.1584259	0.02751
*Eif4e*	Eukaryotic translation initiation factor 4E	1.142376	0.030993
Oxidative stress related genes			
*Gsta5*	Glutathione S-transferase Yc2 subunit	2.4397118	0.013714
**Downregulated**			
Mitocondrial associated genes			
*Mtfr1*	Mitochondrial fission regulator 1	1.2446603	0.047542
*Ptcd3*	Pentatricopeptide repeat domain 3	1.2458737	0.047166
*Inpp5b*	Inositol polyphosphate-5-phosphatase B	1.1909425	0.021694
*Mpv17l2*	MPV17 mitochondrial membrane protein-like 2	1.170515	0.012484
*Bckdha*	Branched chain ketoacid dehydrogenase E1, alpha polypeptide	1.1625147	0.010577
*Pitrm1*	Pitrilysin metallopeptidase 1	1.1204993	0.034489
*Gatm*	glycine amidinotransferase (L-arginine:glycine amidinotransferase)	1.1167421	0.013758
Transcription, translation and metabolism associated genes			
*Gcgr*	Glucagon receptor	1.7509662	0.013298
*Glp2r*	Glucagon-like peptide 2 receptor	1.3274169	0.010548
*Stat6*	Signal transducer and activator of transcription 6	1.2245467	0.041221
Oxidative stress related genes			
*Nrf1*	Nuclear respiratory factor 1	1.1174673	0.039902

Selection of relevant genes from the Agilent Microarray analysis from animals fed with KD compared to CD fed animals. Table shows the mitochondrial related genes; transcription, translational metabolism genes; and oxidative stress associated genes for which expression significantly changed (p < 0.05) (with the exception of *Coq3* that with a p = 0.0554 was included in the selection as the gene we believe is relevant for the study).

Other interesting changes in gene expression were nuclear-coded genes linked to fatty acid and lipid metabolism such *Mttp*, *Fads6* and *Pank*1, or genes associated with glucose homeostasis such *Gcgr* and *Glp2r* (both mRNA levels decreased). Furthermore, increasing mRNAs that encoded for transcription (*Polr2f*, *Tceal1*) or translation (*Eif4e*) factors were found. In addition, genes that contribute to the cell defense mechanisms against oxidative stress such *Gsta5* (up) and *Nrf1*, (down) were also altered by KD.

## Discussion

The beneficial effects of KD in ameliorating the metabolic deficits associated with some neurological disorders are widely recognized and accepted^58,59^. Ketone bodies can interact with specific receptors and trigger the activation of cell signaling pathways that have been shown to play important roles in models of stroke, Alzheimer’s, Parkinson’s disease and Multiple Sclerosis^60^.

Our respirometry data show that KD rescues complex I and II driven O_2_ flux in the injured spinal cord, increases the total protein content of complexes I, II and III in tissue homogenates, and increases cytochrome c oxidase (or complex IV) activity at 7 days post injury. In fact, KD and low-carbohydrate diets have been shown to upregulate expression and activity of components of the mitochondrial ETS complexes^3^, which together with our microarray data and other results presented here suggest that modulation of transcription and the subsequent protein upregulation are linked to a rescue in respiratory function after KD treatment.

Ketone bodies preserve the function of mitochondrial complexes after Traumatic Brain Injury (TBI)^33^, in Purkinje cells in aged rats^61^, in a cellular model of mitochondrial Encephalomyopathy^62^, and after a moderate T9-T10 spinal cord contusion in mice^38^. Moreover, TBI decreases mitochondrial content due to an imbalance between mitochondrial biogenesis and mitophagy that in turn leads to deficiencies in energy metabolism^63^. Our results show a trend towards an increase in the mRNA levels for the mitochondrial biogenesis regulator PGC-1α, and an increase in protein content for the mitochondrial biogenesis marker VDAC1 after SCI in the animals treated with KD. Surprisingly, at the 2 weeks after injury time-point, the data shows a major increase in PCG-1α in both treated groups, suggesting that the injury itself might lead to a compensatory mechanism in which a delayed increase in mitochondria biogenesis might compensate for an initial energetic deficiency. The implications of this compensation, and if this increase in mRNA is truly translated into protein stills to be determined. In regards to a proposed increase in mitochondrial biogenesis by KD, KD increases mitochondrial mass in other neurological disorders such as epilepsy^64^ and ischemic stroke^65^; and recent publications highlight how mitochondrial biogenesis could be a potential pharmaceutical target to treat SCI^66,67^. VDAC1 has also been linked to an increase in the transcription of multiple ETS complexes by interacting with TOM20, a component of the receptor complex responsible for the recognition and translocation of cytosolically synthesized mitochondrial pre-proteins or enzymes required to promote the activation of the transcriptional machinery of the mitochondria^68^. This suggests that an increase in VDAC1 might concurrently promote the expression of ETS complexes. However, it should be noted that the relationship between respiratory function and markers of mitochondrial biogenesis is not always linked^69,70^. Interestingly, our histological analysis using TOM20, a different mitochondrial marker, showed that the injury itself increases mitochondria density in neurons in the intermediate grey/ventral horn area -similarly to what others have shown^71^. However, there were no differences in the expression between CD and KD in neurons indicating that they are unlikely to contribute to the overall changes in mitochondrial content we observed in tissue homogenates after KD treatment. Although with a generally low mitochondrial content compared to other mammalian cells^72^, inflammatory cells might be linked to changes in mitochondrial content after neurotrauma. However, our observations suggest that they would not be major contributors to the increase in mitochondrial density in our model. Indeed, growing evidences suggest that KD or ketones might reduce inflammation^73^, implying that a reduction in mitochondrial content after our treatment should have been expected. It remains to be determined whether (1) other cells (i.e. inflammatory cells, oligodendrocytes or astrocytes) in the lesion site (and vicinities) or/and increase in tissue sparing would show differences and lead to changes in mitochondrial density; (2) if the improvements in mitochondrial respiration that we observed in the injured cord may be partially associated with a global increase in total amount of mitochondria, or (3) what seems more likely, that activation of several signaling pathways involved in the regulation of nuclear and mitochondrial transcription and translational in multiple cell types, as discussed further below.

Mitochondrial DNA (mtDNA) encodes for 13 subunits of the complexes composing the oxidative phosphorylation system (OXPHOS) or ETS^74^. Yet, mitochondria require nuclear DNA coding the remainder of constituent proteins^75^. Extracellular signal‐regulated kinase 1/2 (ERK1/2) is a serine/threonine kinase implicated in signal transduction that is activated in response to growth factors and other intercellular messengers^76^. Interestingly, ERK1/2 can regulate mitochondrial function^77–79^. In HeLa cell cultures, activation of ERK1/2 leads to its interaction with VDAC1 in the mitochondrial outer membrane promoting its translocation into the mitochondrial matrix. Once inside the mitochondrial matrix activated ERK1/2 interacts with the mitochondrial specific transcription factor TFAM in order to promote the transcription of the ETS complexes^40^. Our results show that KD treatment induces an increase in ERK1/2 phosphorylation in spinal cord tissue homogenates, and TFAM expression in tissue homogenates and isolated mitochondria. It is conceivable, that after activation, ERK1/2 is translocated into the mitochondria via VDAC1 to bind to TFAM. However, this mechanism was not fully supported in this study as we were unable to detect ERK1/2 in the mitochondrial fraction. Since TFAM is a nuclear encoded gene^80^, one possibility is that an increased availability of TFAM would also increase its translocation into the mitochondria. Hence, an increase in ERK1/2 activation (and translocation) together with an increase in mitochondrial TFAM could enhance the transcription of some of the ETS complexes in order to compensate for the deficits observed after injury.

Similarly to ERK1/2, several studies have shown that STAT3 can also be found inside the mitochondria (mitoSTAT3) where it regulates the activity of the ETS^81–86^. For example, ciliary neurotrophic factor (CNTF) enhances respiratory capacity, membrane potential and complex IV activity in sensory neurons via STAT3 signaling^87,88^, and cultured astrocytes lacking STAT3 show decreased mitochondrial function^81^. However, the precise mechanism by which STAT3 locally modulates mitochondrial function is unknown. Interestingly, some findings suggest that STAT3 can directly interact with complexes I and II to regulate their activity^84^. Our results indicate that KD treatment increases total STAT3 content—but not the relative phosphorylation levels at Ser727- in tissue homogenates. Interestingly, KD increases the amount of phosphorylated STAT3 (Ser727) in isolated mitochondria, which could be due to an increase in total STAT3 in the tissue homogenate driving the observed increase in mitoSTAT3 that would in the end contribute to an improvement in mitochondrial bioenergetics. Clearly, the efficient function of the ETC requires complex layers of regulation (mitochondria-to-nucleus communication and vice versa) to coordinate the expression of the different subunits, including a combination of transcriptional coordination, sub-cytoplasmic localization of translation and the intricate assembly of the ETC enzyme complexes^89,90^.

KD was found to inhibit the mTOR pathway in liver and brain^42^. mTORC1 regulates intracellular signaling associated with protein synthesis and acts as a nutrient/energy sensitive regulator through, for example, activation of p70-S6K, a serine/threonine kinase that via S6 ribosomal phosphorylation leads to protein synthesis; and inhibition of 4E-BP11, a member of the family of translation repressors^91,92^. Interestingly, our data indicate that a 7 day KD treatment after injury had no effects on PI3 kinase/Akt-dependent mTOR activation (Ser 2448), but increased mTOR total protein content. Although our findings do not support an increase in activation through KD, we cannot preclude that other epitopes such as the Ser2481 (autophosphorylation) could still be phosphorylated and active^93^. Interestingly, we observed different results between the two downstream effectors: p70-S6K and 4E-BP11. KD increased the phosphorylation of p70-S6K after injury but did not affect 4E-BP11 phosphorylation levels. Several mechanisms such as association/dissociation of mTOR associated proteins or post-translational modifications on mTORC1 have been considered as possible mechanisms to explain such differential effects in regulating its downstream effectors^94,95^. mTOR and p70S6K are also found in mitochondria^96,97^. Similar to our observations in tissue homogenates, we found no differences in mTOR phosphorylation at Ser2448 after injury or between treatment groups, but on the contrary there was an increase in total mTOR and p70-S6K phosphorylation in the mTOR mitochondrial pool after injury, both of which are reduced after KD treatment. This reduction in total mTOR and phospho-p70-S6K in the mitochondrial pool after KD might contribute to a reduction of ROS production as previously described^98^, while the increase in the mTOR cytosolic pool might contribute to an increase in nutrient-related protein translation^99^.

Two possible mechanisms could explain the differences in activities and expression of proteins and kinases observed when comparing our tissue homogenate samples to our samples from isolated mitochondria; the first one is the differential rate of kinase degradation by mitochondrial proteases, thus some kinases might have been degraded faster than others^100^; and the second is that some of those kinases might have been removed from the mitochondria after the initial stimulation and downstream signaling activation as previously shown^101^. It is increasingly recognized that proteins and kinases can be translocated to or associating with mitochondria, modulating their differential expression in the mitochondrial pools versus the cytosolic pools (i.e. faster degradation, different activation mechanisms, and different activity levels)^102^. The detailed mechanisms on how these kinases function or are interconnected after SCI or KD treatment would require further study.

Secondary damage after SCI is associated with an increase in oxidative stress^10,103,104^. An overproduction of ROS leads to deficits in enzymatic activity, increase in lipid peroxidation, activation of apoptosis and activation of the antioxidant response, which, together contribute to deficits in mitochondrial bioenergetics after trauma^105^. Similarly to a recently published work from Lu et al.^106^, our data indicate that KD treatment decreases lipid peroxidation (4-HNE) and protein nitration (3NT) at the center of the lesion -where most of the infiltrated inflammatory cells are localized, and activates the NRF2-dependent antioxidant pathway after SCI in vivo* and *in vitro*.* In response to stimuli, NRF2 detaches from KEAP1 in the cytosol and translocates into the nucleus to bind to the antioxidant response element (ARE) leading to the expression of antioxidant-associated genes^107^. Furthermore, NRF2 increases the mitochondrial membrane potential and ATP levels, the rate of respiration and the efficiency of oxidative phosphorylation^108,109^. Indeed, NRF2 plays a key role in regulating the balance between mitochondrial function and the redox state of the cells by modulating the utilization of substrates for mitochondrial respiration (NADH and FADH2), which also appears to be important for the function of antioxidant enzymes^110^. Our data from isolated mitochondria show that KD treatment also induces NRF2 activation. NRF2 can associate with the mitochondria through a complex with KEAP1 and the mitochondrial outer membrane serine/threonine kinase PGAM5. Interestingly, PGAM5 interacts with the antiapoptotic protein BCL-XL^111^ suggesting that KD, through an increase in NRF2 might also be important in reducing apoptosis after SCI. In addition, ROS production changes the activity of TCA cycle enzymes involved in the regulation of cellular metabolism. In skeletal muscle aconitase (AC) undergoes oxidative modification and inactivation after oxidative stress^54,112^. Interestingly the opposite has been reported in the CNS where AC activity is increased at 7 and 28 days after optic nerve transection in rats^113^. Similarly, our results show increased AC activity after SCI at 7 DPI which was reduced by KD treatment. Hence, after SCI KD treatment could decrease AC activity, increase citrate production and thus protect the mitochondria from more oxidative damage leading to recovery of mitochondrial metabolism. These experimental differences also suggest that TCA enzyme function may differ between tissues or after different types of insults.

Finally, our microarray data are in line with previous work from the brain^114^, and as briefly commented at the beginning of the discussion, supports the hypothesis that KD treatment after injury might overall regulate the transcriptional and translational machineries (i.e. *Eif4e, Mrps14, Nfyb*) that control the expression of proteins encoding subunits of some of the complexes (i,e *Ndufa12*), or proteins required for their assembly (i.e. *Cox11*); which would contribute to the bioenergetic rescue after SCI. Additionally, our microarray data also indicate that KD might promote activation of antioxidative stress pathways complementary to the NRF2 pathway, seen by the up- and down-regulation of *Gsta5* and *Nrf1* respectively; and it implies that KD treatment triggers changes in expression of genes encoding proteins with roles in the glucose-fatty acid metabolism as previously reported^114,115^.

**Figure 6:**
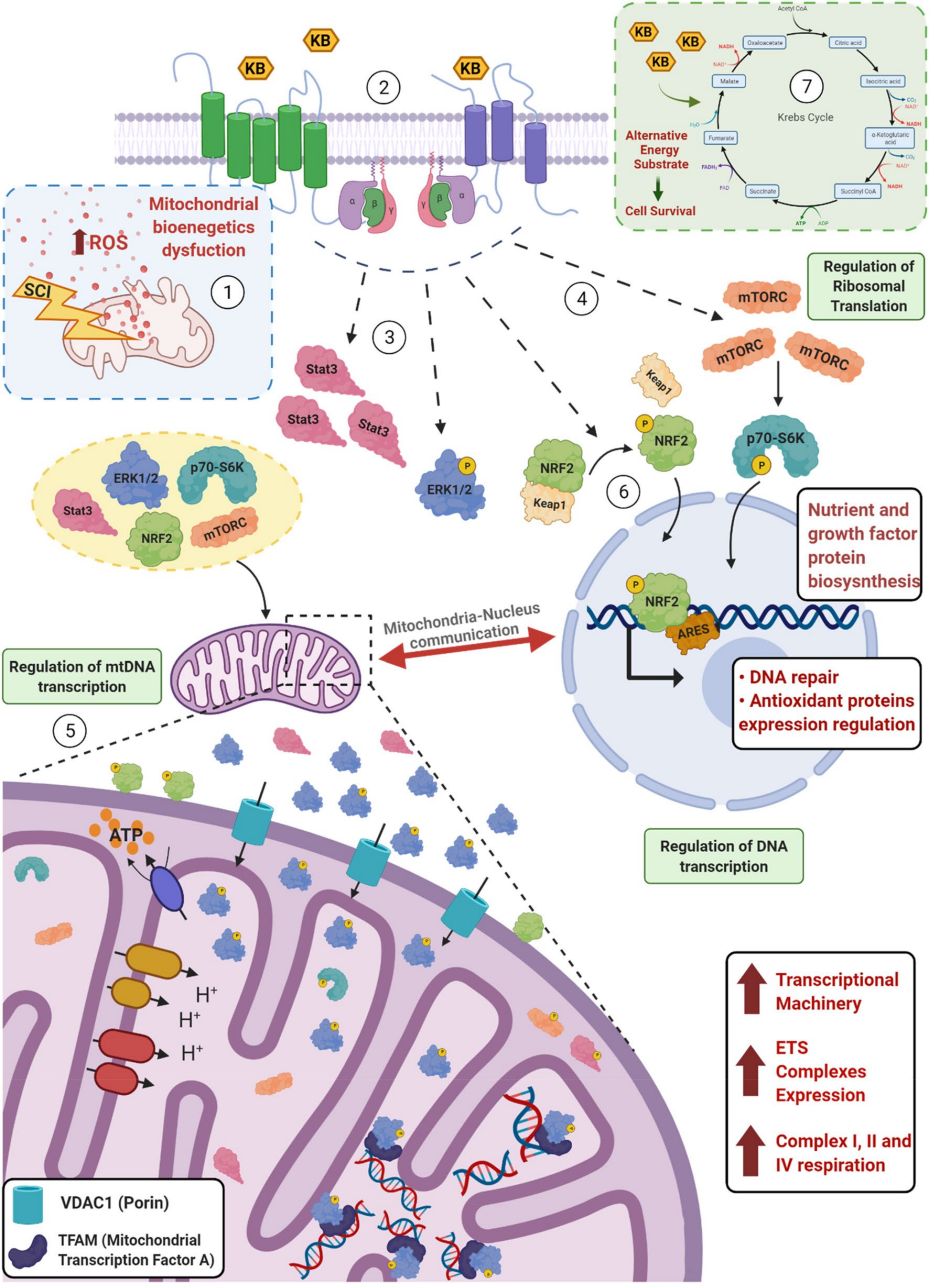
Graphical summary of the proposed mechanism of mitochondrial bioenergetics rescue and activation of antioxidant pathways by ketogenic diet after SCI. After SCI, an increase in ROS production would affect several complexes of the Electron Transport System (ETS) leading to a global dysfunction in cellular bioenergetics. Treatment with KD would lead to an increase in ketone bodies that would bind to specific receptors (i.e. HCA2, FFR2 or unknown). The activation of these receptors would trigger phosphorylation of ERK1/2 and the increase in expression of Stat3 and mTOR . mTOR would regulate ribosomal translation and the subsequent synthesis of proteins and growth factors that are nutrient-dependent. Simultaneously, an increase in VDAC1 expression, might contribute to the translocation of phospho-ERK1/2 (and other essential proteins) into the mitochondrial matrix where by binding the mitochondrial transcription factor (TFAM) would lead to an increase mtDNA transcription and a subsequent expression of several components of the ETS contributing to the mitochondrial bioenergetics rescue. Furthermore, the presence of the mitochondrial pools of Stat3 and mTOR/p70-S6K would contribute to the activation of some of the complexes of the ETS and to reduce ROS production inside the mitochondria. KD treatment would also increase activation of the NRF2-dependent antioxidant pathway that not only would activate expression of antioxidant and DNA repair genes, but whose mitochondrial-associated pool might also help reducing cellular apoptosis. Moreover, KBs might act as an alternative substrate and promote neuroprotection. Overall, our data indicates that KD treatment regulates mtDNA transcription, DNA transcription and ribosomal translation, which altogether contributes to the improvement of mitochondrial bioenergetics after SCI. *Created with BioRender.com.*

In conclusion, our findings—see Fig. 6 for graphical summary- indicate that KD mitigates mitochondrial oxidative damage and dysfunction in acute SCI. Furthermore, we provide new insights into the possible underlying regulatory mechanisms through which KD ameliorates mitochondrial bioenergetics after spinal cord injury (Fig. 6). Additional studies are necessary in order to evaluate whether these early metabolic changes are long lasting or if a long term exposure to ketone bodies, either via KD or ketone esters, might be necessary to sustain these early beneficial effects in cell metabolism seen after SCI.

## Methods

### Experimental design and SCI

All procedures involving animals were conducted according to the guidelines of the Canadian Council for Animal Care and were approved by the University of British Columbia Animal Care Committee. We used 66 adult male Sprague–Dawley rats (~ 300 g at time of injury; Harlan Breeding Laboratory), group-housed (21 °C; 12 h: 12 h light:dark cycle) and given ad libitum access to control rodent diet prior to the injury. The injury was performed as we described previously in JoVE^116^. After a unilateral C5 laminectomy, the dorsal processes of C4-C6 were held with a clamp and fixed in a frame tilted at a 22.5° angle. The contusion force was set to 150 Kdynes using the Infinite Horizon impactor.

### Micro-array

#### RNA extraction

Gene expression 7 days after SCI surgery was chosen to identify and characterize changes between CD (n = 4) and KD (n = 4) treated rats. Total RNA was isolated from 5-mm segment centered on the site of impact according to the instructions of the supplier using the miRvana miRNA isolation kit (Ambion). RNA quantity and integrity were assessed by (Nanodrop ND-1000, Thermo Scientific, USA) and microfluidics-based electrophoresis (Agilent 2100 Bioanalyzer, Agilent Technologies, USA), respectively. RNA samples with OD 260/280 of approximately 2.0 and RIN ≥ 8.0 were used for microarray experiments.

#### cRNA labeling and hybridization to microarrays

Whole-genome gene expression profiles of SCI rats treated with a CD (n = 4) or KD (n = 4) were measured using a SurePrint G3 Rat GE 8 X 60 K Microarray ((G4858A-028279, Agilent Technologies, Palo Alto, CA). A total of 200 ng of total RNA was used and labeled using the Low Input Quick Amp Labeling Kit, One-Color (Agilent Technologies) with Cy3. The microarray was hybridized with the targets for 17 h at 65 °C using a Gene Expression Hybridization Kit (Agilent Technologies), washed using Gene Expression Wash Buffers Pack (Agilent Technologies), and scanned by a Microarray Scanner (G2565CA, Agilent Technologies). All steps were performed according to the manufacturer (Agilent Technologies).

#### Data analysis

The data analysis was performed following the methodology previously described by Oliveira et al.^117^. The Feature Extraction Software v9.1.3.1 (Agilent Technologies) was used to extract and analyze the assay signals and subsequently determine the signal-to-noise ratios from the microarray images. Microarray raw data (.txt files) were imported into R v. 3.0.1 (Team RDC, 2012) and analyzed with the Bioconductor (Gentleman et al., 2004) packages Agi4 × 44PreProcess and limma (Smyth, 2005). Briefly, after quality check, the microarray probes were filtered and their median foreground intensity was normalized within and between arrays according to Agi4 × 44Preprocess and limma user guides, respectively. Finally, the probes were tested for differential expression using a linear model followed by Bayes moderated *t-Student test* (Smyth, 2005) for the comparisons of interest. Genes with nominal *p* < 0.05 were accepted to be differentially expressed. The data was deposited in the NCBI Gene Expression Omnibus (GEO, http://www.ncbi.nlm.nih.gov/geo/) under accession number GSE159555. Genes whose absolute fold change was > 1.1 were selected in Table 1.

### RNA isolation and quantitative real-time polymerase chain reaction (Q-PCR)

A 5 mm segment of the ipsilateral side of the spinal cord centered on the impact site was removed for extraction of RNA. Total RNA was extracted using a Trizol Reagent (Invitrogen, CA, USA) according to the manufacturers’ manual. Quantitative PCR reactions were performed using the TaqMan Fast Universal PCR Master Mix Kit (Applied Biosystems, CA, US)^35^. The mRNA for peroxisome proliferator-activated receptor-gamma coactivator 1α (PGC-1α) was measured by real-time quantitative RTPCR using PE Applied Biosystems prism model 7700. Triplicate reactions were run for each sample for both the gene of interest and the endogenous control (glyceraldehyde-3-phosphate dehydrogenase; GAPDH). Results are presented as 2-dCt, where dCt was difference between cycle threshold (Ct) values of GAPDH and gene of interest, a calculation that compensates for loading errors. Subtraction of dCt of CD rats from dCt of KD rats gives the ddCt value that was used to calculate relative expression levels in KD animals (2-ddCt) as previously described^35^.

### Mitochondrial isolation, protein extraction and western blot analysis

The ipsilateral side of the injured spinal cord segments (5 mm) were dissected out after 7 DPI and homogenized in ice-cold lysis buffer containing 1 × protease inhibitor cocktail and phosphatase inhibitors. Mitochondria were isolated using the Mitochondria Isolation Kit (Biochain. Catalog# KC010100, Newark, CA). The protein concentration from the tissue homogenates and isolated mitochondria was determined using a BCA protein assay kit (Pierce). The extracted proteins were analyzed in polyacrylamide-SDS gels and electro-blotted using PVDF membranes. Some of the membranes were cut prior incubation with the antibodies. After blocking, the membranes were probed overnight at 4ºC with the rabbit anti-phospho-p44/42 MAPK (ERK1/2) (Thr202/Tyr204), anti-Total OXPHOS Antibody Cocktail, anti-p44/42 MAPK (ERK1/2), anti-STAT3, anti-STAT3-Ser727, anti-VDAC1, anti-phospho-NRF2, anti-TFAM, anti-mTOR, anti-phospho-mTOR (Ser2448), anti-phospho-p4E-BP1, anti-Actin, anti-phospho-p70-SK6. Secondary fluorescent antibodies IRDye (800, 680CW IgG (H + L) (1:15,000, LI-COR) were applied to the membrane and incubated for 1 h at room temperature. Blots were visualized using Odyssey Fc Imaging System (LI-COR, Nebraska, USA). Densitometric analysis was performed using Image Studio Lite Ver 5.2. For more detailed information about the antibodies used, see *Table of Antibodies* (Table 2) at the end of the “Methods” section.

**Table 2:** List of antibodies, vendors and used dilutions.

Table of antibodies			
Antibody	Vendor	Catalog Number	Dilution
Phospho-p44/42 MAPK (ERK1/2) (Thr202/Tyr204)	Cell Signaling	9101	1:500
Total OXPHOS Antibody Cocktail	Abcam	ab110413	1:1000
p44/42 MAPK (ERK1/2)	Cell Signaling	9102	1:500
STAT3	Santa Cruz Biotechnology	sc-8019	1:500
STAT3-Ser727	Cell Signaling	9134S	1:500
VDAC1	Abcam	ab14734	1:1000
mTOR	Cell Signaling	7C10	1:500
Phospho-mTOR Ser 2448	Cell Signaling	D9C2	
Phospho-p4E-BP1	Cell Signaling	9459S	1:500
Phospho-p70 S6	Cell Signaling	9205S	1:500
Actin	Proteintech	60,008.1	1:10,000
Tubulin	Abcam	T8660	1:5000
p21	Santa Cruz Biotechnology	sc-6246	1:300
TOM20	Sigma-Aldrich	WH0009804M1	1:300
3NT	Millipore	05-233	1:250
NF200	Sigma-Aldrich	N0142	1:500
4HNE	Calbiochem	393207	1:400
NeuN	Millipore	ABN90P	1:500
TFAM	Santa Cruz Biotechnology	sc-166965	1:500
Phospho-NRF2	Abcam	ab76026	1:1000

### In vitro cultures, treatment, immunocytochemistry and immunohistochemistry

Cortical neurons from C57BL/6 P0–P2 mouse pups were dissociated by combined trypsinization. Cells were placed in 24-well tissue culture dishes (Nunc, Roskilde, Denmark) on coated coverslips and grown for 7 DIV in Neurobasal A medium supplemented with N2 and B27. Cultures were treated then for 24 h with H_2_0_2_ (10 μM, Sigma-Aldrich), β-Hydroxybutyrate (BHB) (4 mM, Sigma-Aldrich) alone or a combination of both. Cultures were stained with anti-p21, anti-phospho-NRF2 and anti-β-III-Tubulin, fixed with 4% PFA mounted in Fluoromount (Vector Labs, Burlingame, CA, USA). The immunofluorescence intensities of the randomized confocal (TCS SPII, Leica, Bannockburn, IL, USA) images (n = 5) taken from each well were analyzed. A total of 14 rats were perfused transcardially with PBS, followed by PFA 4%. The harvested spinal cords were post-fixed for 12–24 h and cryoprotected in a PBS-30% sucrose solution. The cords were subsequently snap frozen in tissue freezing media for cryostat cryosectioning (20 μm section thickness). A full set of sections of each harvested cord was stained for each animal (200 μm between sections). After thawing the sections, the following primary antibodies were used against: TOM20, 3NT, 4HNE, NeuN, NF200, CD45, Iba-1, and GFAP. These were applied overnight at room temperature. Secondary antibodies (1:500, Jackson) conjugated to DyLight 594, 488 and 647, respectively, were applied for 2 h at room temperature. Digital images were captured with an Imager M2 microscope (Zeiss, Jena, Germany) (Immunocytochemistry and Immunohistochemistry protocols as per used in our previous publication^118^). For the lipid peroxidation and protein nitration analysis, 2 different sections of the epicenter of the lesion per animal were evaluated. The spline contour of the whole ipsilateral (injured) side of each section and a ROI in the center of the injury were selected for the intensity measurements. NeuN^+^ staining was used to identify neurons in the intermediate gray/ventral horn area, randomized 9–12 NeuN^+^ cells were outlined and assessed for intensity measurements (TOM20) from four different sections per animal rostral and caudal to the injury (intensity values were averaged). The sections selected were the 4 closest sections to the injury where we did not find neuronal loss. Next, 2 different sections where the injury was still present (rostral and caudal, 1000–1200um from epicenter) were used to outline the injury site (GFAP was used as a reference) and the intensities of the different markers (IBA1, CD45, TOM20) were assessed. The images were analyzed using Zen Software (Zeiss). For more detailed information about the antibodies used, see Table 2 at the end of the “Methods” section.

### Enzymatic activities

Tissue homogenates or isolated mitochondria lysates from the epicenter of the injury were used to quantify the activities of complex IV (Cytochrome C oxidase) as well as citrate synthase by spectrophotometry using the Cytochrome C oxidase Kit (Abcam, ab239711) and Citrate Synthase Assay Kit (Sigma, CS0720) respectively. Citrate Synthase activity defined by the rate of change in the linear range per mg of protein in tissue homogenates corresponding to the epicenter of the injury. Unit definition: 1 unit would oxidize 1 μmol of reduced cytochrome c per min at 25ºC and pH 7.2.

### Mitochondrial respiration

Mitochondrial respiration in permeabilized spinal cord segments was measured using high-resolution respirometry (Oroboros Instruments, Innsbruck, Austria). Experiments were performed at 37 °C in respiration medium containing EGTA (0.5 mM), MgCl_2_ (3 mM), K-lactobionate (60 mM), taurine (20 mM), KH_2_PO_4_ (10 mM), HEPES (20 mM), sucrose (110 mM), and BSA (1 g/l), with the addition of mitochondrial substrates and inhibitors (Substrates-Uncouplers- and Inhibitors (SUIT) Protocol) to measure coupled and uncoupled respiration, flux through complex I and II, maximal respiration of the ETS, and cytochrome oxidase (complex IV respiration). Mitochondrial respiration was expressed as weight-specific oxygen flux (pmolO_2_ s^−1^ mg^−1^) (See Supplementary Fig. S1 for an example of a tracing). Chamber oxygen levels were maintained between 240 and 400 nmol ml^−1^ to avoid O_2_ limitation. b routine respiration was first measured, then malate (2 mM), pyruvate (5 mM), and glutamate (10 mM) were added to measure LEAK respiration (respiration due to proton leakage and the circuit of electrons and cations that is not dependent on ATP synthase activity). ADP (2.5 mM) was then added to measure NADH-dependent coupled respiration through complex I, followed by Oligomycin (2.5 μM) to measure LEAK respiration at high membrane potential during inhibition of ATP synthase. Complete, non-physiological uncoupled respiration of the ETS was measured with the titration of carbonyl cyanide-*p*-trifluoromethoxyphenylhydrazone (CCCP) (0.5 + 0.5 μM). After complex I inhibition with rotenone (0.5 μM), succinate (10 mM) was added to measure reduced flavin adenine dinucleotide (FADH_2_)-dependent complex II respiration. Residual oxygen consumption (ROX) attributed to non-mitochondrial respiration was measured with addition of Antimycin A (2.5 μM) to inhibit complex III of the ETS. Finally, ascorbate (2 mM) and *N*,*N*,*N*′,*N*′-tetramethyl-*p*-phenylenediamine dihydrochloride (TMPD) (0.5 mM) were added to measure the isolated respiratory rate of cytochrome oxidase (complex IV respiration) (See Supplementary Fig. S1 for the quantification of the oxygen flux after sequential titration of the components in our SUIT Protocol).

### Statistical analysis

Statistics were analyzed by using the GraphPad Prism version 6.0.0 for Windows (GraphPad Software, San Diego, California USA, www.graphpad.com). The Shapiro- Wilk test was performed to determine the normality of the data. Wherever appropriate, an unpaired Student’s t-test , one-way or two-way ANOVA, followed by Turkey’s or Fisher’s LSD post hoc comparisons test were used to determine differences between group mean values. Each test used is specified in the figure legends. Data are expressed as means ± SEM. The minimum level of statistical significance was set at P < 0.05.

## Supplementary Information

Supplementary Information.

## References

[1] Alizadeh A, Dyck SM, Karimi-Abdolrezaee S (2019). Traumatic spinal cord injury: An overview of pathophysiology, models and acute injury mechanisms. Front. Neurol..

[2] Yun J, Finkel T (2014). Mitohormesis. Cell Metab..

[3] Miller VJ, Villamena FA, Volek JS (2018). Nutritional ketosis and mitohormesis: Potential implications for mitochondrial function and human health. J. Nutr. Metab..

[4] Carri MT, Polster BM, Beart PM (2018). Mitochondria in the nervous system: From health to disease, part II. Neurochem. Int..

[5] Polster BM, Carri MT, Beart PM (2017). Mitochondria in the nervous system: From health to disease Part I. Neurochem. Int..

[6] Raha S, Robinson BH (2000). Mitochondria, oxygen free radicals, disease and ageing. Trends Biochem. Sci..

[7] Bratic I, Trifunovic A (2010). Mitochondrial energy metabolism and ageing. Biochim. Biophys. Acta.

[8] Musatov A, Robinson NC (2012). Susceptibility of mitochondrial electron-transport complexes to oxidative damage. Focus on cytochrome c oxidase. Free Radic. Res..

[9] Nita M, Grzybowski A (2016). The role of the reactive oxygen species and oxidative stress in the pathomechanism of the age-related ocular diseases and other pathologies of the anterior and posterior eye segments in adults. Oxid. Med. Cell. Longev..

[10] Sullivan PG, Krishnamurthy S, Patel SP, Pandya JD, Rabchevsky AG (2007). Temporal characterization of mitochondrial bioenergetics after spinal cord injury. J. Neurotrauma.

[11] Azbill RD, Mu X, Bruce-Keller AJ, Mattson MP, Springer JE (1997). Impaired mitochondrial function, oxidative stress and altered antioxidant enzyme activities following traumatic spinal cord injury. Brain Res..

[12] McEwen ML, Sullivan PG, Rabchevsky AG, Springer JE (2011). Targeting mitochondrial function for the treatment of acute spinal cord injury. Neurotherapeutics.

[13] Volek JS, Fernandez ML, Feinman RD, Phinney SD (2008). Dietary carbohydrate restriction induces a unique metabolic state positively affecting atherogenic dyslipidemia, fatty acid partitioning, and metabolic syndrome. Prog. Lipid Res..

[14] Westman EC (2007). Low-carbohydrate nutrition and metabolism. Am. J. Clin. Nutr..

[15] Taggart AK (2005). (D)-beta-Hydroxybutyrate inhibits adipocyte lipolysis via the nicotinic acid receptor PUMA-G. J. Biol. Chem..

[16] He W (2004). Citric acid cycle intermediates as ligands for orphan G-protein-coupled receptors. Nature.

[17] Norwitz NG, Hu MT, Clarke K (2019). The mechanisms by which the ketone body D-beta-hydroxybutyrate may improve the multiple cellular pathologies of Parkinson's disease. Front. Nutr..

[18] Tang H, Lu JY, Zheng X, Yang Y, Reagan JD (2008). The psoriasis drug monomethylfumarate is a potent nicotinic acid receptor agonist. Biochem. Biophys. Res. Commun..

[19] Tunaru S (2003). PUMA-G and HM74 are receptors for nicotinic acid and mediate its anti-lipolytic effect. Nat. Med..

[20] Soga T (2003). Molecular identification of nicotinic acid receptor. Biochem. Biophys. Res. Commun..

[21] Chen G (2017). AMP010014A09 in Sus Scrofa encodes an analog of G protein-coupled receptor 109A, which mediates the anti-inflammatory effects of beta-hydroxybutyric acid. Cell Physiol. Biochem..

[22] Huang C (2018). The ketone body metabolite beta-hydroxybutyrate induces an antidepression-associated ramification of microglia via HDACs inhibition-triggered Akt-small RhoGTPase activation. Glia.

[23] Fu SP (2015). beta-Hydroxybutyric acid inhibits growth hormone-releasing hormone synthesis and secretion through the GPR109A/extracellular signal-regulated 1/2 signalling pathway in the hypothalamus. J. Neuroendocrinol..

[24] Rahman M (2014). The beta-hydroxybutyrate receptor HCA2 activates a neuroprotective subset of macrophages. Nat. Commun..

[25] Prins ML, Matsumoto JH (2014). The collective therapeutic potential of cerebral ketone metabolism in traumatic brain injury. J. Lipid Res..

[26] Fortier M (2019). A ketogenic drink improves brain energy and some measures of cognition in mild cognitive impairment. Alzheimers Dement..

[27] Taylor MK, Sullivan DK, Mahnken JD, Burns JM, Swerdlow RH (2018). Feasibility and efficacy data from a ketogenic diet intervention in Alzheimer's disease. Alzheimers Dement. (N Y).

[28] Hasan-Olive MM (2019). A ketogenic diet improves mitochondrial biogenesis and bioenergetics via the PGC1alpha-SIRT3-UCP2 axis. Neurochem. Res..

[29] Veyrat-Durebex C (2018). How can a ketogenic diet improve motor function?. Front. Mol. Neurosci..

[30] Zhao Z (2006). A ketogenic diet as a potential novel therapeutic intervention in amyotrophic lateral sclerosis. BMC Neurosci..

[31] Sullivan PG (2004). The ketogenic diet increases mitochondrial uncoupling protein levels and activity. Ann. Neurol..

[32] Pandya JD (2019). Comprehensive profile of acute mitochondrial dysfunction in a preclinical model of severe penetrating TBI. Front. Neurol..

[33] Greco T, Glenn TC, Hovda DA, Prins ML (2016). Ketogenic diet decreases oxidative stress and improves mitochondrial respiratory complex activity. J. Cereb. Blood Flow Metab..

[34] Maalouf M, Sullivan PG, Davis L, Kim DY, Rho JM (2007). Ketones inhibit mitochondrial production of reactive oxygen species production following glutamate excitotoxicity by increasing NADH oxidation. Neuroscience.

[35] Streijger F (2013). Ketogenic diet improves forelimb motor function after spinal cord injury in rodents. PLoS ONE.

[36] Davis LM, Pauly JR, Readnower RD, Rho JM, Sullivan PG (2008). Fasting is neuroprotective following traumatic brain injury. J. Neurosci. Res..

[37] Kong G (2017). The ketone metabolite beta-hydroxybutyrate attenuates oxidative stress in spinal cord injury by suppression of class i histone deacetylases. J. Neurotrauma.

[38] Qian J, Zhu W, Lu M, Ni B, Yang J (2017). D-beta-hydroxybutyrate promotes functional recovery and relieves pain hypersensitivity in mice with spinal cord injury. Br. J. Pharmacol..

[39] Carbognin E, Betto RM, Soriano ME, Smith AG, Martello G (2016). Stat3 promotes mitochondrial transcription and oxidative respiration during maintenance and induction of naive pluripotency. EMBO J..

[40] Galli S (2009). A new paradigm for MAPK: Structural interactions of hERK1 with mitochondria in HeLa cells. PLoS ONE.

[41] Kim J, Guan KL (2019). mTOR as a central hub of nutrient signalling and cell growth. Nat. Cell. Biol..

[42] McDaniel SS, Rensing NR, Thio LL, Yamada KA, Wong M (2011). The ketogenic diet inhibits the mammalian target of rapamycin (mTOR) pathway. Epilepsia.

[43] Kang D, Kim SH, Hamasaki N (2007). Mitochondrial transcription factor A (TFAM): Roles in maintenance of mtDNA and cellular functions. Mitochondrion.

[44] Lo SC, Hannink M (2008). PGAM5 tethers a ternary complex containing Keap1 and Nrf2 to mitochondria. Exp. Cell. Res..

[45] O'Mealey GB (2017). A PGAM5-KEAP1-Nrf2 complex is required for stress-induced mitochondrial retrograde trafficking. J. Cell. Sci..

[46] Strom J, Xu B, Tian X, Chen QM (2016). Nrf2 protects mitochondrial decay by oxidative stress. FASEB J..

[47] Hall ED (2001). Pharmacological treatment of acute spinal cord injury: How do we build on past success?. J. Spinal Cord Med..

[48] Carrico KM, Vaishnav R, Hall ED (2009). Temporal and spatial dynamics of peroxynitrite-induced oxidative damage after spinal cord contusion injury. J. Neurotrauma.

[49] Bao F, Chen Y, Dekaban GA, Weaver LC (2004). Early anti-inflammatory treatment reduces lipid peroxidation and protein nitration after spinal cord injury in rats. J. Neurochem..

[50] Carvalho AN, Firuzi O, Gama MJ, Horssen JV, Saso L (2017). Oxidative stress and antioxidants in neurological diseases: Is there still hope?. Curr. Drug Targets.

[51] Swink TD, Vining EP, Freeman JM (1997). The ketogenic diet: 1997. Adv. Pediatr..

[52] Villeneuve NF, Sun Z, Chen W, Zhang DD (2009). Nrf2 and p21 regulate the fine balance between life and death by controlling ROS levels. Cell Cycle.

[53] Chen W (2009). Direct interaction between Nrf2 and p21(Cip1/WAF1) upregulates the Nrf2-mediated antioxidant response. Mol. Cell.

[54] Larsen FJ (2016). High-intensity sprint training inhibits mitochondrial respiration through aconitase inactivation. FASEB J..

[55] Bulteau AL, Ikeda-Saito M, Szweda LI (2003). Redox-dependent modulation of aconitase activity in intact mitochondria. Biochemistry.

[56] Castro L, Tortora V, Mansilla S, Radi R (2019). Aconitases: Non-redox iron-sulfur proteins sensitive to reactive species. Acc. Chem. Res..

[57] Tretter L, Adam-Vizi V (2000). Inhibition of Krebs cycle enzymes by hydrogen peroxide: A key role of [alpha]-ketoglutarate dehydrogenase in limiting NADH production under oxidative stress. J. Neurosci.

[58] Stafstrom CE, Rho JM (2012). The ketogenic diet as a treatment paradigm for diverse neurological disorders. Front. Pharmacol..

[59] Gano LB, Patel M, Rho JM (2014). Ketogenic diets, mitochondria, and neurological diseases. J. Lipid Res..

[60] Offermanns S, Schwaninger M (2015). Nutritional or pharmacological activation of HCA(2) ameliorates neuroinflammation. Trends Mol. Med..

[61] Balietti M (2010). A ketogenic diet increases succinic dehydrogenase (SDH) activity and recovers age-related decrease in numeric density of SDH-positive mitochondria in cerebellar Purkinje cells of late-adult rats. Micron.

[62] Frey S (2017). The addition of ketone bodies alleviates mitochondrial dysfunction by restoring complex I assembly in a MELAS cellular model. Biochim. Biophys. Acta.

[63] Sen N (2019). Aberrant cell cycle induction is pivotal for mitochondrial biogenesis after traumatic brain injury. Neural Regen. Res..

[64] Bough KJ (2006). Mitochondrial biogenesis in the anticonvulsant mechanism of the ketogenic diet. Ann. Neurol..

[65] Yin J, Han P, Tang Z, Liu Q, Shi J (2015). Sirtuin 3 mediates neuroprotection of ketones against ischemic stroke. J. Cereb. Blood Flow Metab..

[66] Scholpa NE (2019). Pharmacological stimulation of mitochondrial biogenesis using the food and drug administration-approved beta2-adrenoreceptor agonist formoterol for the treatment of spinal cord injury. J. Neurotrauma.

[67] Scholpa NE, Schnellmann RG (2017). Mitochondrial-based therapeutics for the treatment of spinal cord injury: Mitochondrial biogenesis as a potential pharmacological target. J. Pharmacol. Exp. Ther..

[68] Schleiff E, Silvius JR, Shore GC (1999). Direct membrane insertion of voltage-dependent anion-selective channel protein catalyzed by mitochondrial Tom20. J. Cell. Biol..

[69] Rowe GC (2013). Disconnecting mitochondrial content from respiratory chain capacity in PGC-1-deficient skeletal muscle. Cell. Rep..

[70] Granata C, Jamnick NA, Bishop DJ (2018). Principles of exercise prescription, and how they influence exercise-induced changes of transcription factors and other regulators of mitochondrial biogenesis. Sports Med..

[71] Paschon V (2019). VDAC1 is essential for neurite maintenance and the inhibition of its oligomerization protects spinal cord from demyelination and facilitates locomotor function recovery after spinal cord injury. Sci. Rep..

[72] Robin ED, Wong R (1988). Mitochondrial DNA molecules and virtual number of mitochondria per cell in mammalian cells. J. Cell. Physiol..

[73] Yarar-Fisher C (2021). Ketogenic regimens for acute neurotraumatic events. Curr. Opin. Biotechnol..

[74] Taanman JW (1999). The mitochondrial genome: Structure, transcription, translation and replication. Biochim. Biophys. Acta.

[75] Hutchison CA, Newbold JE, Potter SS, Edgell MH (1974). Maternal inheritance of mammalian mitochondrial DNA. Nature.

[76] Chang L, Karin M (2001). Mammalian MAP kinase signalling cascades. Nature.

[77] Masgras I (2017). Absence of neurofibromin induces an oncogenic metabolic switch via mitochondrial ERK-mediated phosphorylation of the chaperone TRAP1. Cell. Rep..

[78] Rasola A (2010). Activation of mitochondrial ERK protects cancer cells from death through inhibition of the permeability transition. Proc. Natl. Acad. Sci. U S A.

[79] Alonso M (2004). Mitochondrial extracellular signal-regulated kinases 1/2 (ERK1/2) are modulated during brain development. J. Neurochem..

[80] Kang I, Chu CT, Kaufman BA (2018). The mitochondrial transcription factor TFAM in neurodegeneration: Emerging evidence and mechanisms. FEBS Lett..

[81] Wegrzyn J (2009). Function of mitochondrial Stat3 in cellular respiration. Science.

[82] Tammineni P (2013). The import of the transcription factor STAT3 into mitochondria depends on GRIM-19, a component of the electron transport chain. J. Biol. Chem..

[83] Meier JA, Larner AC (2014). Toward a new STATe: The role of STATs in mitochondrial function. Semin. Immunol..

[84] Szczepanek K, Chen Q, Larner AC, Lesnefsky EJ (2012). Cytoprotection by the modulation of mitochondrial electron transport chain: The emerging role of mitochondrial STAT3. Mitochondrion.

[85] Gough DJ (2009). Mitochondrial STAT3 supports Ras-dependent oncogenic transformation. Science.

[86] Sarafian TA (2010). Disruption of astrocyte STAT3 signaling decreases mitochondrial function and increases oxidative stress in vitro. PLoS ONE.

[87] Chowdhury SR (2014). Ciliary neurotrophic factor reverses aberrant mitochondrial bioenergetics through the JAK/STAT pathway in cultured sensory neurons derived from streptozotocin-induced diabetic rodents. Cell. Mol. Neurobiol..

[88] Luo X (2016). Enhanced transcriptional activity and mitochondrial localization of STAT3 co-induce axon regrowth in the adult central nervous system. Cell. Rep..

[89] Sirey TM, Ponting CP (2016). Insights into the post-transcriptional regulation of the mitochondrial electron transport chain. Biochem. Soc. Trans..

[90] Tang JX, Thompson K, Taylor RW, Olahova M (2020). Mitochondrial OXPHOS biogenesis: Co-regulation of protein synthesis, import, and assembly pathways. Int. J. Mol. Sci..

[91] Dazert E, Hall MN (2011). mTOR signaling in disease. Curr. Opin. Cell. Biol..

[92] Saxton RA, Sabatini DM (2017). mTOR signaling in growth, metabolism, and disease. Cell.

[93] Peterson RT, Beal PA, Comb MJ, Schreiber SL (2000). FKBP12-rapamycin-associated protein (FRAP) autophosphorylates at serine 2481 under translationally repressive conditions. J. Biol. Chem..

[94] Choo AY, Blenis J (2009). Not all substrates are treated equally: Implications for mTOR, rapamycin-resistance and cancer therapy. Cell Cycle.

[95] Choo AY, Yoon SO, Kim SG, Roux PP, Blenis J (2008). Rapamycin differentially inhibits S6Ks and 4E-BP1 to mediate cell-type-specific repression of mRNA translation. Proc. Natl. Acad. Sci. U S A.

[96] Harada H, Andersen JS, Mann M, Terada N, Korsmeyer SJ (2001). p70S6 kinase signals cell survival as well as growth, inactivating the pro-apoptotic molecule BAD. Proc. Natl. Acad. Sci. U S A.

[97] Ramanathan A, Schreiber SL (2009). Direct control of mitochondrial function by mTOR. Proc. Natl. Acad. Sci. U S A.

[98] Miwa S (2016). Decreased mTOR signalling reduces mitochondrial ROS in brain via accumulation of the telomerase protein TERT within mitochondria. Aging (Albany NY).

[99] Meier JA (2017). Stress-induced dynamic regulation of mitochondrial STAT3 and its association with cyclophilin D reduce mitochondrial ROS production. Sci. Signal..

[100] Quiros PM, Langer T, Lopez-Otin C (2015). New roles for mitochondrial proteases in health, ageing and disease. Nat. Rev. Mol. Cell. Biol..

[101] McLelland GL, Soubannier V, Chen CX, McBride HM, Fon EA (2014). Parkin and PINK1 function in a vesicular trafficking pathway regulating mitochondrial quality control. EMBO J..

[102] Lim S (2016). Regulation of mitochondrial functions by protein phosphorylation and dephosphorylation. Cell Biosci..

[103] Visavadiya NP, Patel SP, VanRooyen JL, Sullivan PG, Rabchevsky AG (2016). Cellular and subcellular oxidative stress parameters following severe spinal cord injury. Redox Biol..

[104] Jia Z (2012). Oxidative stress in spinal cord injury and antioxidant-based intervention. Spinal Cord.

[105] Huang ML (2019). The role of the antioxidant response in mitochondrial dysfunction in degenerative diseases: Cross-talk between antioxidant defense, autophagy, and apoptosis. Oxid. Med. Cell. Longev..

[106] Lu Y (2018). Ketogenic diet attenuates oxidative stress and inflammation after spinal cord injury by activating Nrf2 and suppressing the NF-kappaB signaling pathways. Neurosci. Lett..

[107] Nguyen T, Huang HC, Pickett CB (2000). Transcriptional regulation of the antioxidant response element. Activation by Nrf2 and repression by MafK. J. Biol. Chem..

[108] Dinkova-Kostova AT, Baird L, Holmstrom KM, Meyer CJ, Abramov AY (2015). The spatiotemporal regulation of the Keap1-Nrf2 pathway and its importance in cellular bioenergetics. Biochem. Soc. Trans..

[109] Kovac S (2015). Nrf2 regulates ROS production by mitochondria and NADPH oxidase. Biochim. Biophys. Acta.

[110] Baird L, Dinkova-Kostova AT (2011). The cytoprotective role of the Keap1-Nrf2 pathway. Arch. Toxicol..

[111] Lo SC, Hannink M (2006). PGAM5, a Bcl-XL-interacting protein, is a novel substrate for the redox-regulated Keap1-dependent ubiquitin ligase complex. J. Biol. Chem..

[112] Lushchak OV, Piroddi M, Galli F, Lushchak VI (2014). Aconitase post-translational modification as a key in linkage between Krebs cycle, iron homeostasis, redox signaling, and metabolism of reactive oxygen species. Redox Rep..

[113] Cummins N (2013). Changes to mitochondrial ultrastructure in optic nerve vulnerable to secondary degeneration in vivo are limited by irradiation at 670 nm. BMC Neurosci..

[114] Noh HS (2004). A cDNA microarray analysis of gene expression profiles in rat hippocampus following a ketogenic diet. Brain Res. Mol. Brain Res..

[115] Cullingford TE, Eagles DA, Sato H (2002). The ketogenic diet upregulates expression of the gene encoding the key ketogenic enzyme mitochondrial 3-hydroxy-3-methylglutaryl-CoA synthase in rat brain. Epilepsy Res..

[116] Lee JH (2012). A contusive model of unilateral cervical spinal cord injury using the infinite horizon impactor. J. Vis. Exp..

[117] de Oliveira GP, Alves CJ, Chadi G (2013). Early gene expression changes in spinal cord from SOD1(G93A) Amyotrophic Lateral Sclerosis animal model. Front. Cell Neurosci..

[118] Seira O, Liu J, Assinck P, Ramer M, Tetzlaff W (2019). KIF2A characterization after spinal cord injury. Cell. Mol. Life Sci..

