# Supplementary material for: Ketogenesis controls mitochondrial gene expression and rescues mitochondrial bioenergetics after cervical spinal cord injury in rats
Source: Sci Rep. 2021 Aug 11;11:16359. doi: 10.1038/s41598-021-96003-5 (PMC8357839; doi:10.1038/s41598-021-96003-5)
Supplement: Supplementary file 1 — Supplementary Information. [file 41598_2021_96003_MOESM1_ESM.docx]

***Title: Ketogenesis controls mitochondrial gene expression and rescues mitochondrial bioenergetics after cervical spinal cord injury in rats.***

**Authors:** Oscar Seira, Kathleen Kolehmainen, Jie Liu, Femke Streijger, Anne Haegert, Stéphane Lebihan, Robert Boushel and Wolfram Tetzlaff


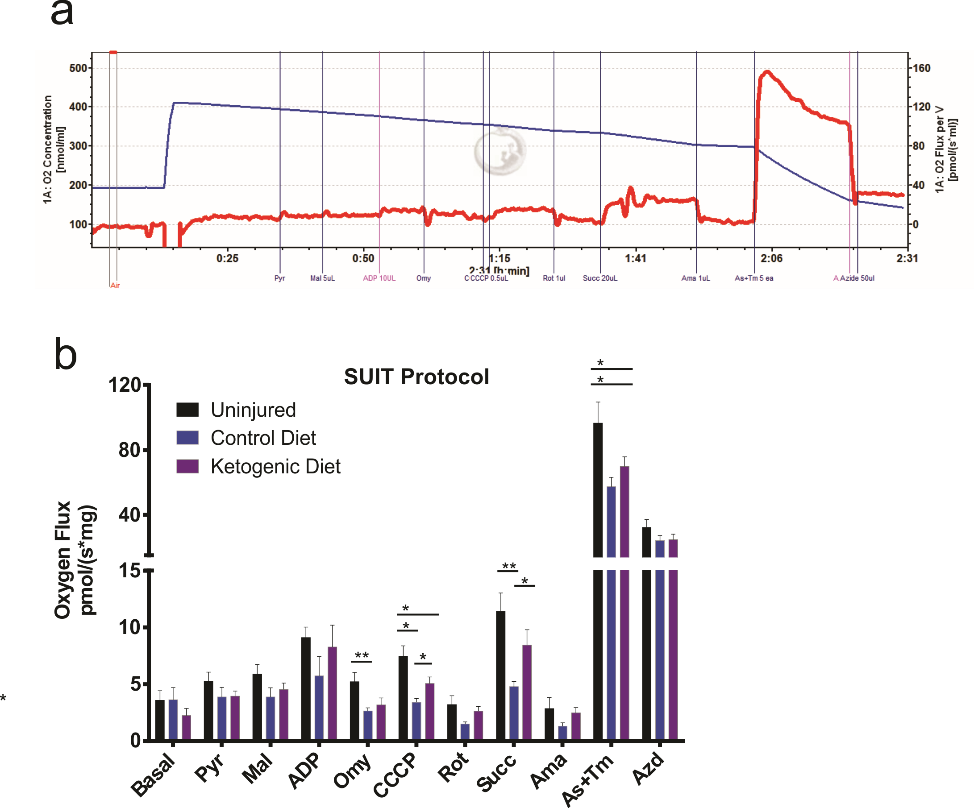


**Supplementary Figure 1. Tracing from the Oroboros High Resolution Respirometry and experimental SUIT Protocol. a)** Example of an uninjured high resolution respirometry tracing. The Y axis on the left represents the Oxygen concentration in nmol/ml (blue line) and the Y axis on the right represents the Oxygen flux in pmol/(s*mg) (red line). The X axis represents the time in hours, and each of the titration steps is represented by a vertical line with its abbreviated name at the bottom. **b)** Oxygen Flux plot of the different titrations sequentially organized (SUIT Protocol). Studedn’t t-tests.


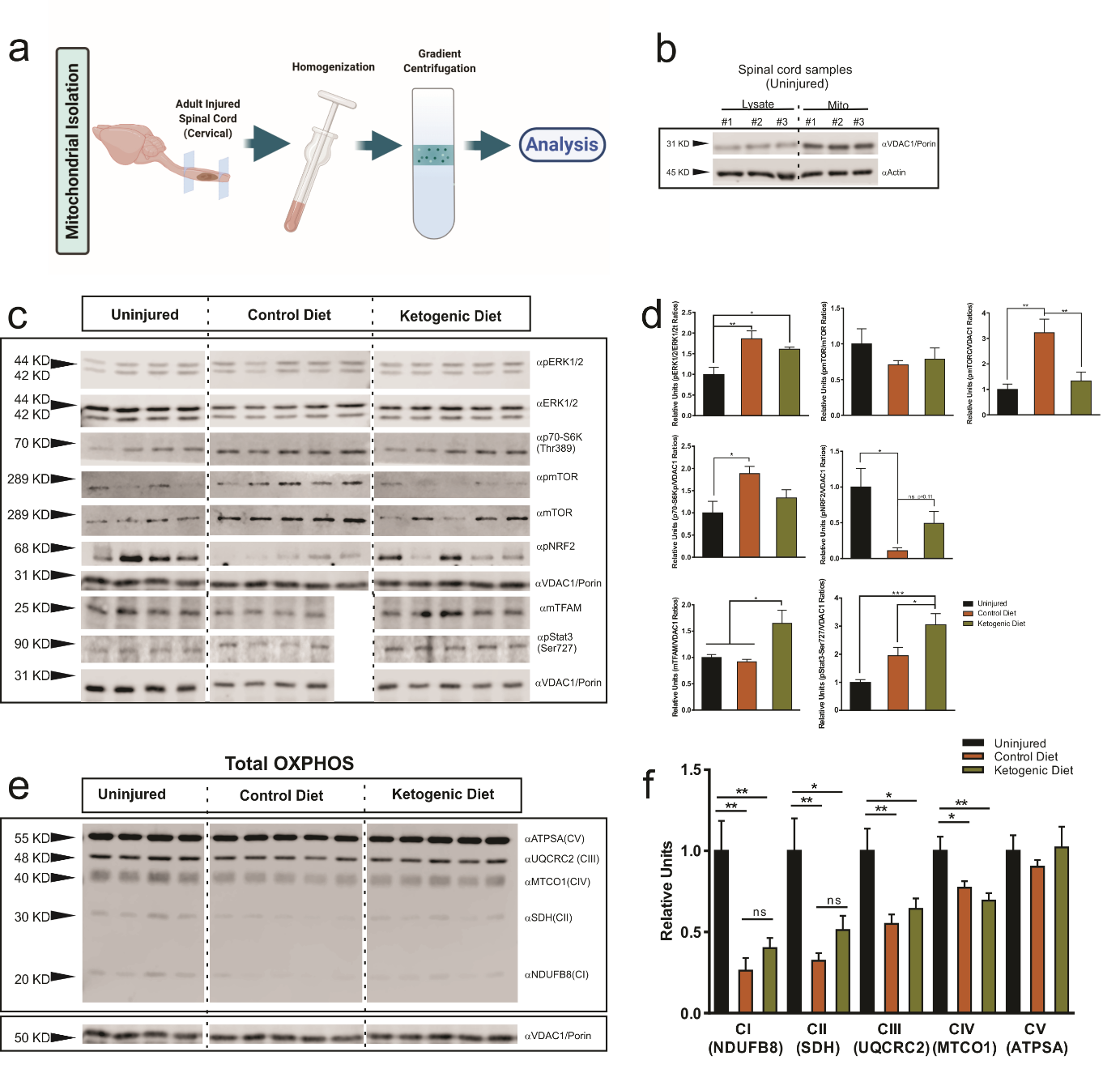


**Supplementary Figure 2. Western blot analysis of mTFAM, NRF2, and members of the MAPK, mTOR and STAT signaling pathways in isolated mitochondria from spinal cords after KD treatment. a)** Graphical representation of the mitochondria isolation protocol. *Created with BioRender.com.* **b)** Enriched mitochondrial content after isolation (VDAC1 expression) compared to regular tissue homogenates. **c)** Analysis of expression and phosphorylation of proteins of the MAPK, mTOR and STAT signaling pathways from uninjured spinal cord and injured cords treated with control (CD) or ketogenic diet (KD). **d-l)** Quantitative analysis of (c). **m-n)** Isolated mitochondrial extracts were subjected to SDS-PAGE followed by western blot analysis using the total OXPHOS antibody cocktail. *The blots from the same gels were cropped and rearranged in the following order: Uninjured, Control Diet, Ketogenic Diet; to make them more clear and enhance reader’s understanding of the figure and the subsequent quantifications. Due to the low yield of mitochondria after isolation, and the use of same antibody hosts in the OXPHOS blot, the same VDAC1 blot had to be used in that analysis after running out of protein samples. Full-length blots/gels are presented in Supplementary Figure 5. One-way ANOVA, Fisher’s LSD Post-hoc test.* *All data are mean ± SEM.*

**
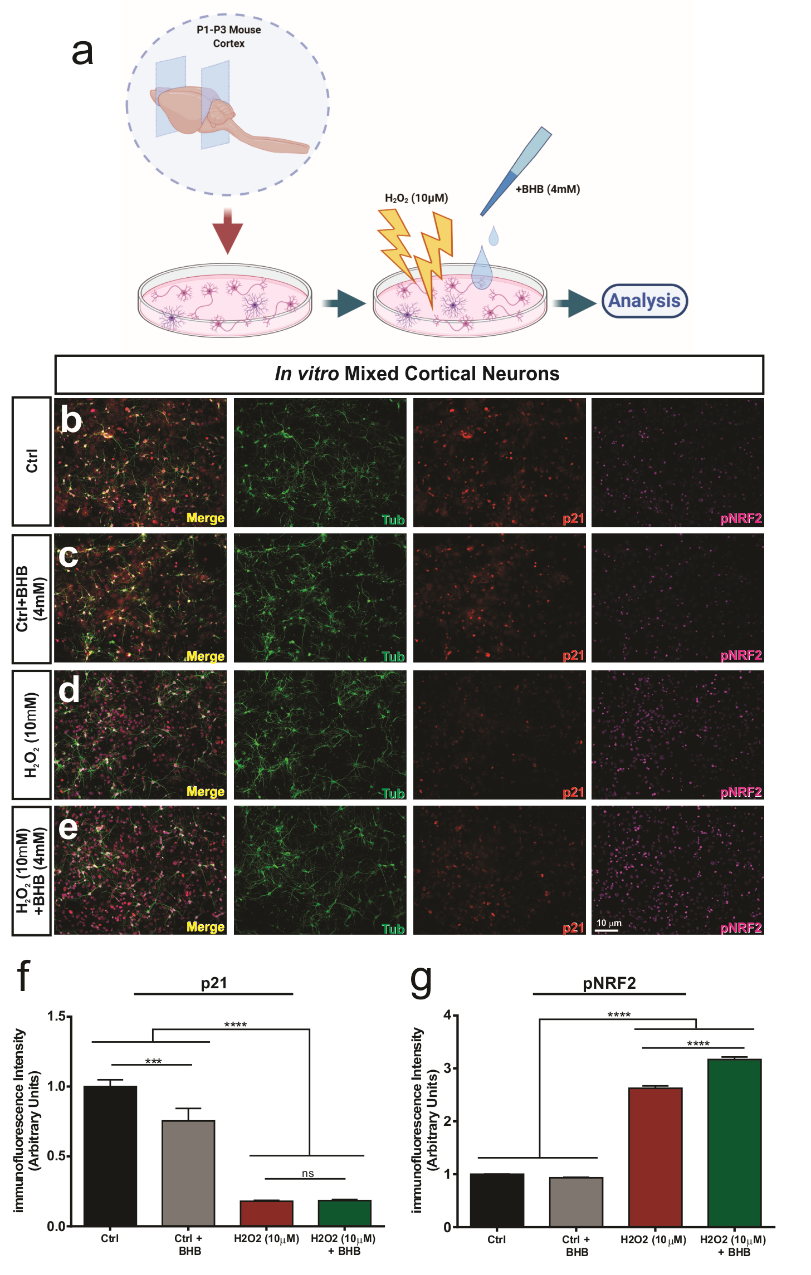
**

**Supplementary Figure 3. Effect βHB treatment on oxidative stress *in vitro*. a-g)** Response of mixed cortical cultures to H_2_O_2_ and β-hydroxybutyrate (BHB). Schematic representation of the *in vitro* treatment protocol. *Created with* [*BioRender.com*](https://biorender.com/)*.*

**b-e)** Representative micrographs of mixed cortical cultures stained with anti-p21 (red), phospho-NRF2 (pink) and anti-tubulin (green) under the different treatment conditions. Image (b) showing a control image where the cultures were not treated. In image (c) cortical neurons were treated with 4mM of BHB, and finally mixed cultures were treated with H_2_O_2_ with or without addition of BHB (a and e respectively). **f-g)** Quantification of the intensities (p21 and pNRF2) from randomized regions of interest for each of the cultures were quantified. We observed an overall increase in the intensity levels for the antioxidant transcription factor pNRF2 after H_2_O_2_ treatment that was significantly higher in the H_2_O_2_ + BHB group compared to the H_2_O_2_ alone. p21 intensity levels drastically dropped after treatment with H_2_O_2_. *One-way ANOVA, Fisher’s LSD Post-hoc test. Values are shown as mean ± SEM* (n=3 different cultures from 2 independent experiments)*.*

**
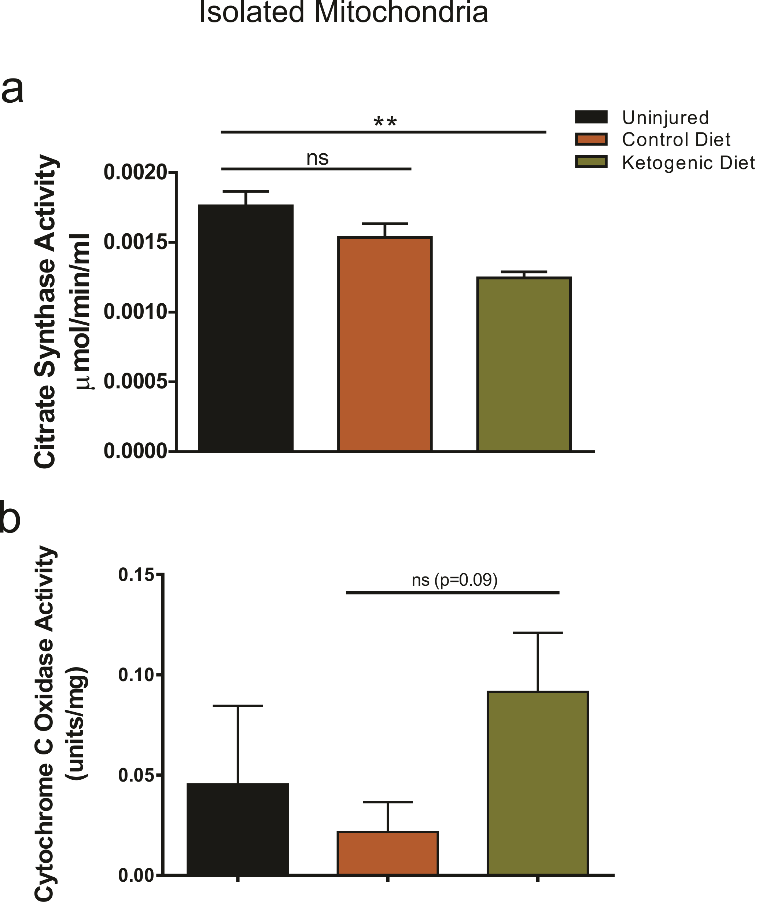
**

**Supplementary Figure 4. Citrate synthase (CS) and Cytochrome C Oxidase activities in isolated mitochondria. a-b)** Citrate synthase y and Cytochrome C oxidase activities measured in mitochondria isolated from the injury site (uninjured, n=4; control diet, n=5; ketogenic diet, n=5). *One-way ANOVA, Fisher’s LSD Post-hoc test. Values are shown as mean ± SEM.*

*
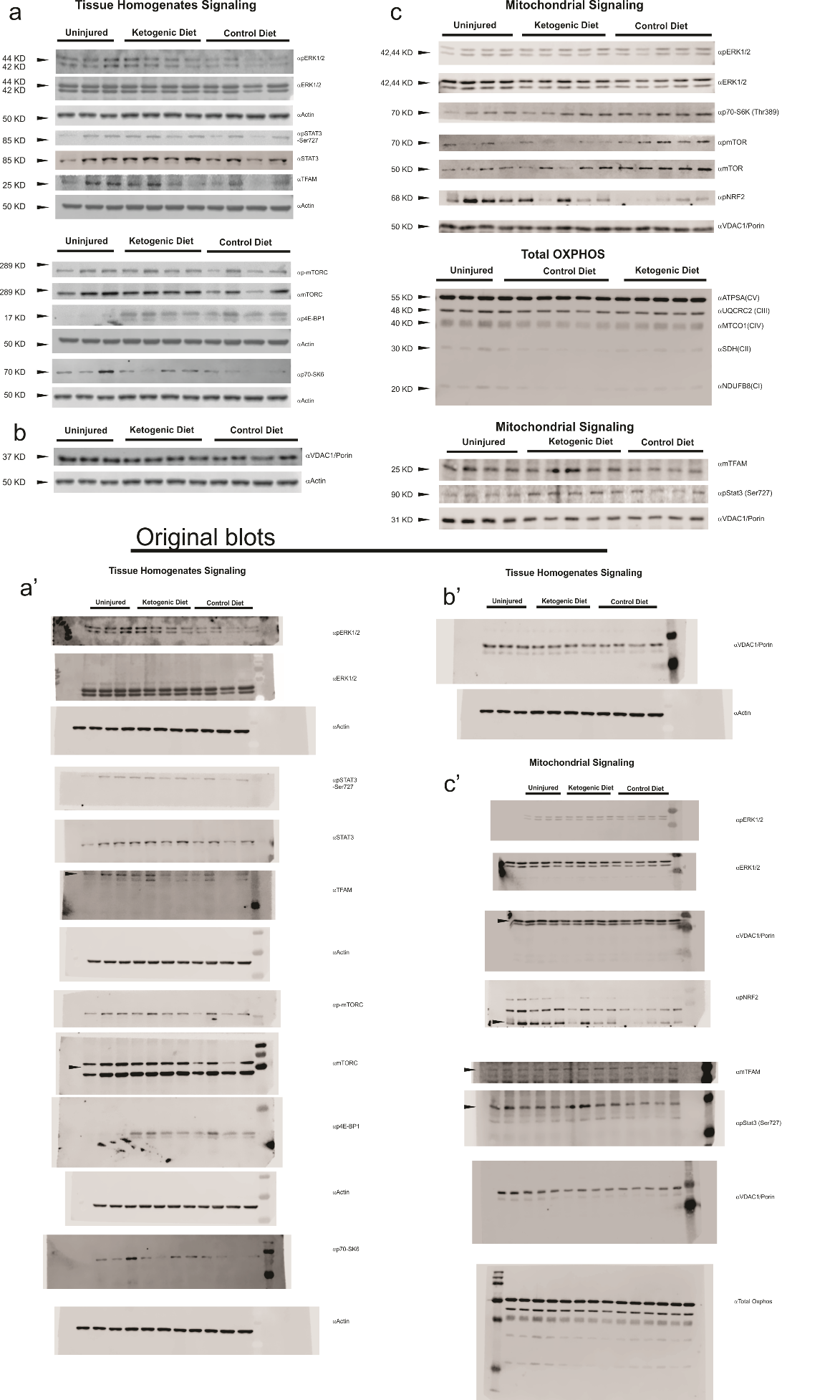
*

**Supplementary Figure 5. Full length blots/gels and originals (‘). a, a’)** Blots correspond to blots shown in Figure 4a and 4c. Most of the membranes that are cropped were cut previous incubation with primary antibodies in order to optimize limited sample use. **b, b’)** Blots shown in Figure 3a. **c, c’)** Blots shown in Supplementary Figure 4c and 4e. The original blots for mTORC and phospho-mTORC were not available.
